# Training needs of informal caregivers of persons with dementia in rural southwestern Uganda: A qualitative formative study

**DOI:** 10.1017/gmh.2026.10279

**Published:** 2026-07-30

**Authors:** Joyce Namwase, Julius Kyomya, Amos Oyuru, Emmanuel Maleka, Abraham Muhwezi, John Semuwemba, Christine K. Karungi, Celestino Obua, Edith K. Wakida

**Affiliations:** 1 Psychiatry, Mbarara University of Science and Technology Faculty of Medicine, Uganda; 2 Psychiatry, Butabika National Referral Mental Hospital, Uganda; 3 Pharmacy, Mbarara University of Science and Technology Faculty of Medicine, Uganda; 4 Pharmaceutical Science, Mbarara University of Science and Technology, Uganda; 5 Community Health, Mbarara University of Science and Technology, Uganda; 6 Physiology, Mbarara University of Science and Technology, Uganda; 7 Public Health, Bishop Stuart University, Uganda; 8 Alpha Center of Research and Administration, Mbarara University of Science and Technology, Uganda; 9 Medical Education, California University of Science and Medicine School of Medicine, USA

**Keywords:** informal caregivers, dementia, training needs, rural Uganda, low-resource settings

## Abstract

Dementia is a global public health concern with increasing prevalence, especially in low- and middle-income countries (LMICs) where care is primarily provided by informal family caregivers. This study identified the specific training needs of informal caregivers of persons with dementia in rural southwestern Uganda to inform the development of contextually appropriate caregiver training interventions. We conducted 20 in-depth interviews with 17 caregivers and 3 key informants affiliated with Reach One Touch One Ministries (ROTOM), a community-based organization supporting older persons in Rukiga District, Uganda. Interviews were audio-recorded, transcribed verbatim, and analyzed thematically using an inductive approach. Caregivers demonstrated limited knowledge of dementia, often attributing it to normal aging, spiritual causes, or stress. They faced substantial physical, emotional, and financial burdens, and relied on faith, social support, and adaptive caregiving strategies. All expressed willingness to attend group-based, in-person training focused on practical skills, including communication, managing dementia-related behaviors, personal hygiene, nutrition, and medication administration. Key informants highlighted the importance of community awareness to address misconceptions. Informal caregivers in rural Uganda experience high caregiving burden and unmet training needs. The identified training priorities closely align with World Health Organization (WHO) iSupport domains, supporting adaptation of the program for rural Ugandan caregivers.

## Graphical abstract

**Figure:**
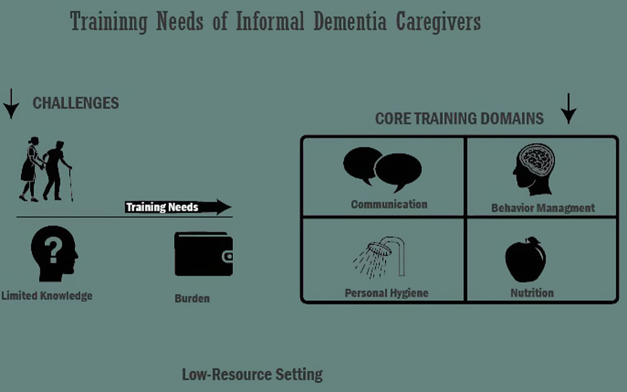

## Impact statement

Dementia is a public health concern with increasing prevalence, especially in low- and middle-income countries (LMICs). The burden of caring for persons living with dementia largely falls on informal caregivers, who are usually family members with little or no formal training or support. Informal caregivers play a central role in caring for persons with dementia in rural Uganda, yet their specific training needs are rarely documented or incorporated into intervention design. This study provides evidence from caregivers and health providers, highlighting critical gaps in dementia knowledge, caregiving skills, and psychosocial support. The findings have practical implications for the development of culturally appropriate, low-cost caregiver training interventions in resource-limited settings. Priority areas identified include communication, nutrition, hygiene, behavior management, and caregiver self-care. The study provides guidance for adapting evidence-based tools, including the World Health Organization’s (WHO’s) iSupport tool, to rural African contexts. Addressing these needs has the potential to improve caregiver well-being, reduce burden of caregiver, and enhance the quality of care provided to persons living with dementia. The study contributes to global mental health by highlighting the perspectives of informal caregivers in low-resource settings and underscoring the importance of context-specific intervention design. The findings may inform policymakers, program implementers, and non-governmental organizations in developing community-based dementia care strategies and integrating dementia care into primary and community health services. Strengthening caregiver capacity is a critical step toward more equitable and sustainable dementia care globally.

## Background

Dementia is an umbrella term for several diseases that affect memory, other cognitive abilities and behavior that interfere significantly with a person’s ability to maintain their activities of daily living (World Health Organization, 2017). Globally, more than 55 million people were living with dementia by 2020, a number projected to nearly double every 20 years, reaching 78 million by 2030 and 139 million by 2050 (Prince et al., 2013, Alzheimerʼs Disease International, n.d.). Currently, approximately 60% of people with dementia live in LMICs, and this proportion is projected to increase to 71% by 2050 (Prince et al., 2013). In sub-Saharan Africa, the number of persons living with dementia is projected to increase threefold from 2015 to 2050 (Prince et al., 2017). In Uganda, the prevalence of dementia among individuals 60 years and above in southwestern regions is estimated at 20% (Mubangizi et al., 2020). This high prevalence, particularly in rural LMIC settings, is associated with substantial strain on healthcare systems that are often characterized by limited resources and restricted access to specialized care.

As a result, responsibility for dementia care is often pushed to family members, commonly referred to as “invisible second patients” because of the substantial burden they experience (Brodaty and Donkin, 2009; Engel et al., 2022). In sub-Saharan Africa, caregivers of persons with dementia frequently experience significant physical, emotional, social, and financial challenges, including stress, depression, social isolation, disrupted family and occupational roles, and strained interpersonal relationships as caregiving responsibilities increasingly demand continuous supervision and support (Brodaty and Donkin, 2009; Komuhangi et al., 2022; Montgomery et al., 2023; Owokuhaisa et al., 2023). In some settings, psychosocial stress and caregiver burden have also been associated with negative caregiving outcomes, including neglect and abusive behaviors toward persons living with dementia (Cooper et al., 2010; Batista-Malat et al., 2025; Hernandez Chilatra et al., 2026). Limited dementia-related knowledge, inadequate caregiving skills, and restricted access to formal psychosocial support services further contribute to caregiver distress and may negatively affect the quality of care provided (van den Kieboom et al., 2020; Wang et al., 2022). Despite these challenges, caregivers employ a variety of coping mechanisms to manage caregiving stress and sustain long-term caregiving roles. Studies from sub-Saharan Africa have documented coping strategies including empathy and perspective-taking, family support and cohesion, humor and positive communication, emotional support seeking, spirituality and religious coping, acceptance, and other culturally informed approaches that foster resilience and adaptation among caregivers (Owokuhaisa et al., 2023; Duodu et al., 2024). Evidence further suggests that caregivers predominantly rely on emotion-focused coping strategies, particularly religious coping, acceptance, and emotional support seeking, while problem-focused strategies such as planning and dysfunctional coping approaches such as self-distraction are used less frequently (Owokuhaisa et al., 2023). However, despite reliance on these informal coping mechanisms, access to structured caregiver training, respite care, and formal psychosocial support services remains limited in many low-resource settings.

In Uganda, dementia care remains relatively underdeveloped, and there is limited dementia-specific policy guidance or structured caregiving support within the healthcare system (Kakongi et al., 2020). Dementia services are largely delivered through general mental health, non-communicable disease, and community-based care services, with most caregiving responsibilities falling on informal family caregivers due to limited specialized geriatric and dementia care services (Ainamani et al., 2020b). Although Uganda has made substantial progress in integrating palliative care into the national healthcare system through hospital-, community-, and home-based models (Kagarmanova et al., 2022), dementia-specific palliative and caregiver support services are still emerging and remain less well described within the literature. Consequently, caregivers of people living with dementia often receive inadequate training, psychosocial support, and guidance on coping with caregiving-related stress (Abaasa et al., 2023). Recent work in Uganda has demonstrated the feasibility and acceptability of culturally adapting the WHO iSupport program for dementia caregivers, highlighting emerging efforts to integrate structured caregiver support interventions into the healthcare system (Gumikiriza-Onoria et al., 2024). These findings underscore the need to strengthen evidence-based caregiver support interventions within Uganda’s healthcare and palliative care system.

In response to these challenges, the WHO developed the iSupport tool, an online and offline self-help and training intervention designed to provide education, skills training, and psychosocial support for dementia caregivers (World Health Organization, 2019). The program consists of structured modules and lessons that offer practical guidance on dementia care and caregiver well-being. Its content covers understanding dementia, managing behavioral and psychological symptoms, communication strategies, daily caregiving skills, stress management, self-care, and accessing social and community support. The tool is intended for use by informal caregivers, healthcare providers, and community-based support programs as a structured educational and psychosocial intervention aimed at strengthening caregivers’ knowledge, coping abilities, and practical caregiving skills. Through these components, the program is expected to enhance caregivers’ capacity to manage the physical, emotional, and behavioral needs of people living with dementia, improve the quality of care provided, reduce caregiver stress and burden, and promote caregivers’ mental health and overall well-being (World Health Organization, 2019).

The WHO-iSupport program has been culturally adapted across diverse settings (Baruah et al., 2021; Omachi et al., 2023; Turana et al., 2023). These adaptations have included translation into local languages, modification of caregiving examples to fit local cultural contexts, and adjustment for both online and offline delivery based on available resources and levels of digital access. In some settings, the program has also been integrated into community-based caregiver support initiatives and primary healthcare services to improve accessibility among informal caregivers. Studies from these adapted settings have reported improvements in caregiver knowledge, coping skills, psychological well-being, and reduced caregiver burden (Baruah et al., 2021; Gumikiriza-Onoria et al., 2024). In Uganda, the program has similarly demonstrated acceptability and potential effectiveness among caregivers of people living with dementia in central Uganda (Gumikiriza-Onoria et al., 2024).

Despite growing evidence supporting caregiver-focused tools and interventions, caregivers of persons with dementia in rural southwestern Uganda remain largely underserved, with limited access to structured training or support (Owokuhaisa et al., 2023). While caregiver burden and psychosocial outcomes have been documented in Uganda, there is limited empirical evidence detailing what caregivers themselves identify as priority training needs to inform contextually appropriate caregiver training interventions (Ainamani et al., 2020a; Owokuhaisa et al., 2023). Understanding caregiver-defined training priorities is a critical step in adapting evidence-based tools, such as WHO iSupport, for rural, low-resource settings. Against this background, this study aimed to identify the training needs of informal caregivers of persons with dementia and to guide the development of a caregiver training intervention using the WHO iSupport program in rural Uganda.

## Methods

### Study design and setting

This was a qualitative, cross-sectional formative study conducted in October 2024 to determine the specific training needs of informal caregivers of elderly persons with dementia in rural Uganda. The study involved informal caregivers and key informants from Reach One Touch One Ministries (ROTOM), a not-for-profit organization that provides support to the elderly in southwestern Uganda.

The study was conducted in Muhanga Town Council in Rukiga District, Kigezi sub-region in southwestern Uganda. According to the 2014 National Population and Housing Census, Kigezi has a substantial older population, accounting for approximately 6% of Ugandaʼs population aged 60 years and above based on census estimates (Uganda Bureau of Statistics, 2016). More recent census data indicate that Rukiga District had an estimated population of 132,355 in 2024, of whom 11,494 (8.7%) were aged 60 years and above (Uganda Bureau of Statistics, 2024). ROTOM was established in 2003 and supports vulnerable older persons through a range of services, including food assistance, clothing, psychosocial support groups, and healthcare services. Its health center provides outpatient and inpatient care, laboratory services, physiotherapy, medical outreaches, health screening, and home-based care, among others. Beneficiaries access services either at the facility or through community outreach visits conducted by ROTOM workers (Reach One Touch One Ministries, n.d.). This study was conducted and reported in accordance with the Consolidated Criteria for Reporting Qualitative Research (COREQ) guidelines (Supplementary File S2).

### Study population, sample size, and sampling

The study population comprised primary informal caregivers of persons living with or suspected to have dementia who were receiving support through ROTOM dementia care activities. Most caregivers were family members, although one non-family caregiver providing unpaid care in a home setting was also included in the study. Additionally, key informants included healthcare professionals and staff members affiliated with ROTOM who were directly involved in dementia care delivery, caregiver support, or community outreach activities.

Seventeen informal caregivers and three key informants were purposively recruited. Data collection and analysis were conducted concurrently. For caregiver interviews, the stopping criterion was saturation, which was considered achieved when successive interviews no longer generated new codes, concepts, or themes related to caregivers’ experiences and coping strategies. Saturation was assessed through ongoing review and comparison of interview transcripts during the data collection process. For key informant interviews, participant numbers were determined purposively based on the diversity of professional roles and their direct involvement in dementia care delivery and caregiver support, rather than saturation alone.

Informal caregivers were purposively selected from the ROTOM caregiver database to ensure inclusion of participants with direct experience caring for persons living with or suspected to have dementia. Selection considered variation in caregiver age, gender, and duration of caregiving experience to capture diverse perspectives relevant to dementia caregiving in rural Uganda.

Potential participants were identified from the ROTOM caregiver registry and contacted by trained research assistants, with support from ROTOM staff, to introduce the study and provide information regarding its purpose and procedures. Individuals who expressed interest in participating were invited to take part in the study, and interview appointments were subsequently scheduled via mobile telephone communication at times and locations convenient to the participants. Key informants were purposively selected based on their professional roles and direct involvement in dementia care delivery, caregiver support, and community outreach activities within ROTOM.

### Data collection tools

We developed two interview guides; one for informal caregivers and the other for key informants. The guides were informed by the WHO iSupport framework (World Health Organization, 2019) and existing literature on challenges faced by informal dementia caregivers (Ainamani et al., 2020a, 2020b; Komuhangi et al., 2022; Kamoga et al., 2023; Owokuhaisa et al., 2023). Specifically, questions were informed by core domains of the WHO iSupport framework, including dementia knowledge, caregiving skills, coping strategies, and caregiver support needs. The detailed interview guide is provided as a Supplementary File (Supplementary File S1).

The interview guides were translated into Ruyankore-Rukiga to accommodate participants who were not fluent in English.

Interviews were conducted by two female trained research assistants, each with a bachelor’s degree in Social Sciences and prior training in qualitative data collection. Interviews were conducted by trained research assistants who were experienced in qualitative research, had no prior relationship with participants, and were fluent in both English and Ruyankore-Rukiga. Written informed consent was obtained from all participants, and those who could not write provided thumbprints after the purpose of the study was explained to them. Interviews were conducted at participants’ homes or workplaces, audio-recorded with consent, and anonymized to protect confidentiality. Additionally, field notes were taken during interviews to capture contextual information and nonverbal cues. Interviews lasted approximately 30 to 60 minutes, and we conducted no repeat interviews. Audio files were stored securely in password-protected databases accessible only to the research team

### Data management and analysis

The audio recordings were transcribed verbatim to preserve the richness of the data and translated into English by individuals proficient in both Ruyankore-Rukiga and English. Transcripts were reviewed alongside the original audio files by members of the research team (JK, AM, JN) to ensure accuracy. The research team read and re-read the transcripts to familiarize themselves with the data.

To ensure rigor and trustworthiness, several strategies were employed. Credibility was enhanced through the use of semi-structured interviews, prolonged engagement with participants during interviews, and concurrent data collection and analysis to allow emerging issues to be explored further. Regular discussions among the research team were conducted to review codes, categories, and themes and to ensure consistency in interpretation. Dependability was supported through documentation of the research process and analytic decisions. Reflexivity was maintained by acknowledging the researchers’ backgrounds and potential influence on data interpretation throughout the study process. Transferability was enhanced by providing detailed descriptions of the study setting, participants, and procedures.

A coding framework and coding tree illustrating the evolution of codes into subthemes and themes were developed by JK and reviewed by EKW (Supplementary File S3). To enhance analytic rigor, coding was conducted independently by JN, JK, EM, AM, AO, and JS, and discrepancies were resolved through discussion and consensus. Data were analyzed using Braun and Clarke’s thematic analysis (Braun and Clarke, 2006), combining inductive coding with sensitizing concepts drawn from the WHO iSupport framework. Caregiver and key informant interviews were initially coded separately to allow identification of stakeholder-specific perspectives. During thematic analysis, codes and emerging themes from both participant groups were compared and synthesized to identify areas of convergence, complementarity, and divergence across stakeholder experiences and perceptions. Themes were refined through iterative discussions among the research team to ensure that interpretations reflected both caregiver and key informant perspectives.

Manual coding was conducted using a coding matrix to identify key themes and subthemes. Codes were iteratively refined to ensure alignment with participants’ perspectives.

### Ethics approval and consent to participate

This study was conducted in accordance with the principles of the Declaration of Helsinki. Ethical approval was obtained from the Gulu University Research and Ethics Committee (approval number GUREC-2024-962) and the Uganda National Council for Science and Technology (approval number HS5165ES). All participants provided voluntary written informed consent prior to participation. This study did not involve a clinical trial; therefore, trial registration was not applicable.

## Results

### Participant characteristics

A total of 17 informal caregivers and 3 key informants were interviewed. Most of the caregivers were female (*n* = 13) compared to males (*n* = 4). Their ages ranged from 25 to 75 years. The duration of caregiving by the participants ranged between 2 and 30 years (Table 1).

**Table 1.** Characteristics of informal caregivers for people with dementia in Rukiga district, Uganda, October 2024 (*n* = 17) Table 1. long description.

Participant	Sex	Age (years)	Highest level of education	Relationship to person with dementia	Duration of giving care
P1	Female	40	Primary	Mother	10
P2	Female	48	Primary	Mother	3
P3	Female	54	No formal education	Father-in-law	6
P4	Female	25	Primary	Grand parent	5
P5	Male	75	No formal education	Wife	8
P6	Female	62	Primary	Mother	2
P7	Male	42	Primary	Grand mother	7
P8	Female	40	Secondary	Mother-in-law	6
P9	Female	50	No formal education	Mother	5
P10	Female	66	Primary	Sister	13
P11	Female	60	Primary	Mother	9
P12	Female	58	Primary	Mother-in-law	3
P13	Female	60	Primary	Mother	13
P14	Male	36	No formal education	Mother	24
P15	Female	75	No formal education	Husband	22
P16	Male	30	Secondary	Not related	7
P17	Female	58	Primary	Father	30

Overall, participants represented a heterogeneous caregiving population with wide variation in age, educational attainment, caregiving duration, and relationship to the person with dementia, providing a broad perspective on caregiving experiences and training needs (Table 1).

**Table 2.** Characteristics of key informants on dementia care in Rukiga District, Uganda, October 2024 (*n* = 3) Table 2. long description.

Participant	Gender	Age (years)	Profession	Years of experience
Key informant 1	Female	30	Nurse	11
Key informant 2	Female	28	Clinical officer	1
Key informant 3	Male	31	Medical officer	4

### Themes

The major themes identified from the qualitative interviews include: Knowledge about dementia, caregiving burden and challenges, coping strategies of caregivers, Training needs and preferences, and potential barriers to caregiver training participation. While both caregivers and key informants emphasized limited dementia knowledge and caregiver burden, differences emerged in their perspectives. Key informants more strongly highlighted cultural misconceptions, delayed care-seeking, and medication non-adherence, whereas caregivers focused more on the day-to-day physical, emotional, and financial challenges of caregiving.

Sub-themes are presented under each thematic area to reflect variations in perspectives between caregivers and key informants.

#### Theme 1: Knowledge about dementia

##### Sub-theme: Caregiver understanding of dementia

Most caregivers demonstrated a limited understanding of dementia and often described it in terms of forgetfulness, confusion, and misplacing items. Dementia was commonly perceived as a normal part of aging rather than a medical condition requiring specialized care. Caregivers often recognized changes in memory and behavior but lacked the language or medical understanding to explain these symptoms.

*This condition confuses me because you can be discussing with someone, and within a minute, they have already forgotten what you were talking about* Caregiver 8, Female, 75 years

Key informants similarly observed that dementia symptoms were commonly normalized within communities and therefore not viewed as requiring medical attention.

*… some know that it comes with aging…* Key Informant 2, Female 28 years

These perceptions contributed to low dementia literacy and reduced recognition of dementia as a health condition requiring formal support and management.

##### Sub theme: Causes of dementia

Caregivers were generally unable to identify specific medical causes of dementia. Instead, dementia was attributed to a range of perceived causes including stress, chronic illness, medication use, accidents, grief and spiritual or supernatural factors. Such explanations reflected broader community beliefs and attempts to make sense of progressive cognitive decline in older adults.

*I was thinking that maybe in the family, they worship the devil ….* Caregiver 15, Female, 75 years

Several caregivers also associated dementia with excessive worrying, old age, or long-term suffering, suggesting that emotional distress and life hardship were viewed as contributing to mental deterioration.

##### Subtheme: Beliefs about treatment and care-seeking

The key informants explained that beliefs in witchcraft and spiritual causation strongly influenced help-seeking behaviors among families caring for persons with dementia. In many cases, caregivers sought assistance from traditional healers, churches, or prayer groups before seeking formal medical care.

…. *they normally take dementia as bewitching, they take it as someone who has been bewitched, they don’t know that it is also a disease like the rest of the diseases…* Key Informant 1, Female, 30 years

Some caregivers and families also expressed doubts regarding the effectiveness of biomedical treatment, which contributed to delayed care-seeking and poor medication adherence.

*… you find them not believing in the drug that you are giving them…* Key Informant 3, Male, 31 years

#### Theme 2: Physical, emotional, and financial burden of caregiving

##### Subtheme: Caregiving tasks

The caregivers described dementia caregiving as a demanding and continuous responsibility that structured much of their daily routine. Caregiving activities commonly included feeding, bathing, administering medication, supervising movement, and assisting with toileting. Many caregivers described the need for constant monitoring due to forgetfulness, wandering, or behavioral changes in persons with dementia.

*I wake up and give her medication… I start on lunch….* Caregiver 4, Female 25 years

Caregiving responsibilities frequently limited opportunities to engage in farming, paid work, or other household activities, contributing to economic strain within families.

##### Subtheme: Stress and exhaustion

Caregiving was perceived as physically and emotionally exhausting. Caregivers described experiencing fatigue, emotional distress, financial pressure, and social isolation due to the continuous demands of caregiving and limited support from others.

*I am the only one responsible for her care….* Caregiver 14, Male, 36 years

Several caregivers explained that they had reduced or abandoned previous livelihood activities in order to provide full-time care.

*I no longer do them because of this woman.* Caregiver 5, Male, 75 years

The emotional burden of caregiving was intensified by uncertainty regarding disease progression and the absence of adequate respite or shared caregiving arrangements.

##### Subtheme: Behavioral and supervision challenges

Caregivers described significant challenges related to behavioral symptoms of dementia, including aggression, wandering, disrupted sleep, refusal of medication, repetitive questioning, and inappropriate social behaviors. These behaviors often required constant supervision and increased caregiver stress.

*Sometimes she wakes up and wants to go out and walk naked….* Caregiver 5, Male, 75 years

Difficulties related to hygiene, feeding, and toileting were particularly challenging for caregivers managing advanced stages of dementia. Caregivers also expressed concern about maintaining the dignity and safety of persons with dementia while balancing other family responsibilities. The cumulative physical, emotional, and economic strain described by caregivers underscores the need for training that addresses both practical caregiving skills and caregiver well-being.

#### Theme 3: Coping strategies of caregivers

##### Subtheme: Coping with caregiving challenges

Caregivers described several informal coping strategies used to manage the emotional and practical demands of caregiving. Faith-based coping mechanisms such as prayer, church attendance, and reliance on spiritual support were commonly reported and were often described as sources of emotional comfort and perseverance.

*I asked for God’s grace to help me…* Caregiver 1, Female, 40 years

Some caregivers also relied on patience, emotional restraint, and acceptance in order to maintain caregiving relationships despite ongoing stress.

##### Subtheme: Acceptance and adaptation

Over time, some caregivers described gradually adapting to the caregiving role and accepting behavioral changes associated with dementia. This adaptation was often framed as a sense of obligation, responsibility, or compassion toward the affected family member.

*Over these years, I have accepted to take her as she is.* Caregiver 10, Female, 66 years

Acceptance appeared to help some caregivers sustain long-term caregiving despite limited resources and support.

##### Subtheme: Caregiving isolation and limited support

Although some caregivers reported occasional support from neighbors or relatives, many described caregiving as an isolating experience with little shared responsibility. Several participants expressed feelings of loneliness and helplessness arising from prolonged caregiving demands.

*It is my burden to die with.* Caregiver 5, Male, 75 years

This statement reflected emotional resignation and perceived caregiving isolation rather than a coping strategy itself. Overall, caregivers described limited access to formal psychosocial support, respite care, or structured caregiver assistance.

While caregivers shared common experiences of burden and limited dementia knowledge, variations emerged in coping strategies, caregiving duration, access to family support, and perceived caregiving challenges depending on gender, caregiving role, and available social support.

#### Theme 4: Training needs and preferences

##### Subtheme: Need for training

All caregivers expressed willingness to participate in caregiver training and viewed training as an opportunity to improve caregiving knowledge, skills, and confidence. Caregivers particularly valued opportunities to learn from healthcare workers and other caregivers with similar experiences.

*We learn from other people…* Caregiver 11, Female, 60 years

Many participants believed that training could improve communication with persons with dementia and reduce caregiving difficulties.

##### Subtheme: Training format

Caregivers preferred in-person, group-based training approaches that allowed interaction, practical demonstrations, and shared learning experiences.

*We all learn how to give care to our people.* Caregiver 4, Female, 25 years

Key informants similarly emphasized the importance of participatory training approaches, practical examples, and translated materials that could be easily understood and referenced within the community context.

##### Subtheme: Content desired

Caregivers and key informants identified several priority training areas, including communication skills, management of difficult behaviors, hygiene, nutrition, medication administration, self-care for caregivers and care of bedridden patients.

*There is a lot to learn. Caregiver 3,* Female, 54 years

These priorities reflected caregivers’ desire for practical and contextually relevant skills that could directly improve daily caregiving experiences.

#### Theme 5: Potential barriers to participation in training intervention

##### Subtheme: Time constraints and competing responsibilities

Despite strong interest to attend training, caregivers identified several barriers to participation, including time constraints, transport challenges, continuous caregiving responsibilities, and a lack of alternative care arrangements to provide temporary support.

*If it starts early and ends late… I would fail to attend.* Caregiver 13, Female, 60 years

Many caregivers explained that they had limited personal freedom and found it difficult to leave persons with dementia unattended.

*I don’t have the liberty to take a break.* Caregiver 16, Male, 30 years

These findings highlight the importance of flexible community-based and caregiver-sensitive training models that accommodate caregiving demands and broader social responsibilities.

Overall, the perspectives of caregivers and key informants were largely complementary. Both groups identified limited community understanding of dementia, substantial caregiver burden, and the need for practical caregiver training. However, key informants more explicitly emphasized the influence of cultural beliefs and misconceptions on delayed care-seeking and medication nonadherence, whereas caregivers focused more on the day-to-day physical, emotional, and financial challenges of caregiving. Key informants additionally highlighted the importance of structured and participatory training approaches, while caregivers emphasized peer support, practical demonstrations, and flexibility in training delivery.

## Discussion

This study aimed to identify the specific training needs of informal caregivers as foundational evidence to inform the development of dementia caregiver training interventions in rural Uganda. Our findings reveal a complex caregiver experience characterized by limited dementia-related knowledge, a substantial caregiving burden, and a strong willingness to acquire practical skills. Despite limited formal support, caregivers demonstrated resilience through adaptive coping strategies, underscoring both unmet needs and existing strengths that can be leveraged in intervention design.

### Caregiver understanding of dementia

Our results indicate that most caregivers interviewed in this rural Ugandan setting attributed dementia primarily to normal aging, spiritual attachments, and stress, with limited recognition of its status as a medical condition. These misconceptions, shaped by cultural beliefs and local explanatory models, align with findings from other LMICs where dementia awareness remains low (Ferri and Jacob, 2017; Johnston et al., 2020; Owokuhaisa et al., 2020; Pokala et al., 2025). Similar interpretations of dementia as a non-medical or culturally defined condition have been reported across rural settings, reflecting broader gaps in community health education (Njohjam et al., 2026). Although caregivers recognized common symptoms such as forgetfulness and functional decline, many lacked a biomedical understanding of dementia and its progression. Consequently, these misconceptions appeared to influence how caregivers interpreted symptoms and interacted with formal healthcare services, often contributing to delayed help-seeking and reliance on non-medical sources of support. This finding highlights dementia literacy as a critical target for caregiver training interventions and community awareness programs. The persistence of spiritual and age-related explanations may reflect the absence of accessible dementia education within rural communities, where cognitive decline is often interpreted through existing cultural frameworks rather than biomedical models. Such interpretations may not only delay diagnosis but also influence caregiving expectations, treatment decisions, and the willingness of families to engage with formal health services.

### Caregiving burden and challenges

The informal caregivers reported significant physical, financial and emotional burdens associated with their caregiving roles. The multidimensional burden reported by caregivers mirrors findings from both high- and low-resource settings, suggesting that dementia caregiving challenges may transcend healthcare contexts, although resource limitations may intensify their impact in rural Uganda (Liu et al., 2022; Gumikiriza-Onoria et al., 2024; Martis et al., 2024). In Uganda, high levels of psychological distress and depression among dementia caregivers have been documented, with up to 64.4% experiencing significant mental health challenges (Gumikiriza-Onoria et al., 2024).

Importantly, caregiver burden in this setting appears to be amplified by structural factors including poverty, limited access to health services, and the absence of respite care and formal support systems. Unlike caregivers in many high-income settings who may access specialist services and social support programs, participants in this study often managed caregiving responsibilities with minimal external assistance. This suggests that interventions in rural Uganda should address not only caregiving skills but also broader support needs that influence caregiver well-being.

The absence of structured formal support systems likely exacerbates caregiver burden, particularly in rural settings where health and social services are limited. Challenges related to hygiene management, sleep disruption, financial insecurity, and limited personal time highlight the need for interventions that address both caregiving competence and caregiver well-being, rather than focusing solely on patient care tasks. The findings also suggest that caregiving responsibilities disproportionately fall on women, reflecting broader gendered expectations of informal caregiving within many African family systems.

### Coping strategies of caregivers

Caregivers relied heavily on faith-based practices and informal social support as coping mechanisms, findings that align with earlier research demonstrating the central role of spirituality and community support in stress management among caregivers (Puchalski, 2012; Duodu et al., 2024; Salifu et al., 2025). Faith-based coping appeared to serve multiple functions beyond spiritual comfort. It provided a framework for meaning-making, emotional regulation, and resilience in circumstances where formal psychosocial services were largely unavailable.

While these practices provide emotional comfort, they also point to the absence of formal psychosocial support services for caregivers in this setting. Caregivers described adapting their communication styles and caregiving approaches over time to better respond to the needs of persons with dementia. This intuitive adaptation reflects a degree of emotional intelligence and experiential learning that could be strengthened through structured training. However, limited opportunities for respite, social engagement, and shared caregiving responsibilities raise concerns about caregiver isolation, which has been linked to increased burden and burnout in other studies (Grycuk et al., 2022; Lim and Lee, 2025). These findings reinforce the importance of interventions that promote self-care, peer support, and community-based caregiving networks. Faith-based coping may reflect the central role of religion and community spirituality in emotional resilience within rural Ugandan communities.

The gradual acceptance and adaptation reported by some caregivers suggest the development of experiential caregiving competence over time. While acceptance may reduce emotional conflict and facilitate long-term caregiving, it may also contribute to normalization of caregiver burden if adequate support systems are absent. Training interventions should therefore seek to build on caregivers’ existing adaptive strategies while addressing unmet support needs.

### Training needs and preferences

The strong willingness of caregivers to participate in training represents a critical opportunity to develop interventions. This finding aligns with evidence from caregiver studies demonstrating that tailored educational programs can improve caregiving knowledge, competence, and psychosocial outcomes (Phillips et al., 2016; Yuan et al., 2025). Importantly, caregivers articulated clear, practical training priorities, providing direct guidance for structured caregiver interventions.

Limited understanding of dementia as a medical condition underscores the importance of incorporating foundational education on dementia etiology, progression, and management into training curricula. The emotional and physical burden described by caregivers further supports the inclusion of self-care and stress management strategies, which align with Module 3 of the WHO iSupport tool. Perspectives from key informants reinforced the need for training in communication and behavior management, particularly for addressing challenging behaviors.

Caregivers’ preference for in-person, group-based training reflects the communal nature of rural settings and offers opportunities for peer learning and mutual support. This preference is consistent with evidence from group-based caregiver training models, where shared learning enhances both knowledge acquisition and emotional well-being (Felstead et al., 2023). Overall, the training needs identified closely align with the core domains of the WHO caregiver support tool, supporting its potential for contextual adaptation in rural Uganda (World Health Organization, 2019). Notably, the training priorities identified by participants emerged organically from their lived experiences and closely mirrored the core domains of the WHO iSupport program. This convergence suggests that iSupport provides a potentially suitable framework for adaptation within rural Ugandan communities while also emphasizing the importance of contextual modifications to reflect local language, culture, caregiving practices, and health-system realities.

### Potential barriers to participation in training intervention

The barriers identified by caregivers highlight an implementation challenge frequently encountered in community-based caregiver interventions. High caregiving demands, limited transport, and lack of alternative caregivers may restrict participation even when perceived need is high (Perkins et al., 2022). These barriers were evident in our findings, where caregivers described continuous caregiving demands and restricted personal freedom. Future programs should therefore prioritize flexible delivery models, including shorter sessions, community-based venues, and blended approaches that minimize disruption to caregiving responsibilities. Evidence from pilot studies in Uganda adapting the WHO iSupport tool demonstrates that structured, contextually adapted psychosocial interventions can improve caregiversʼ well-being and quality of life, supporting the feasibility and acceptability of such approaches in similar settings (Gumikiriza-Onoria et al., 2024). These findings are consistent with previous studies showing that caregiver burden is associated with financial strain and caregiver depression, underscoring the need for practical caregiving skills and psychosocial support for families caring for people with dementia (Pokala et al., 2025). However, our findings suggest that the benefits of such interventions can only be fully realized if delivery strategies accommodate caregiversʼ competing responsibilities, mobility limitations, and resource constraints. Taken together, these findings support the use of flexible, community-based approaches to enhance participation and accessibility of caregiver interventions in low-resource settings.

### Implications for policy, practice, and future research

Taken together, these findings suggest that improving dementia care in rural Uganda requires a comprehensive approach that extends beyond increasing caregiver knowledge alone. Effective caregiver support programs should simultaneously address dementia literacy, practical caregiving skills, caregiver well-being, and community-level misconceptions that influence care-seeking behaviors. The combination of high caregiver burden, limited formal support, and strong interest in training indicates a substantial unmet need that may be addressed through culturally adapted caregiver support programs.

At the policy level, the limited understanding of dementia and the absence of structured caregiver support highlight the need to integrate dementia awareness, caregiver education, and psychosocial support into primary healthcare and community-based health programs. Policymakers should consider incorporating caregiver-focused interventions into national mental health, aging, and non-communicable disease strategies, particularly in rural areas where formal support services remain limited.

For practice, the findings support the development and contextual adaptation of structured caregiver training interventions such as the WHO iSupport program. Training should address practical caregiving skills, communication strategies, management of behavioral symptoms, and caregiver self-care. Delivering training through community-based and group-oriented approaches using local languages may enhance accessibility, peer support, and acceptability among caregivers in rural communities.

Future research should evaluate the feasibility, acceptability, and effectiveness of culturally adapted caregiver training interventions in improving caregiver well-being and quality of dementia care. Further studies involving larger and more diverse populations across different regions of Uganda are also needed to better understand contextual differences in caregiving experiences and support needs. Longitudinal and intervention studies may additionally help identify sustainable strategies for reducing caregiver stress and improving dementia care outcomes. Collectively, the findings provide empirical guidance for the cultural adaptation of the WHO iSupport program by identifying locally relevant knowledge gaps, caregiving challenges, preferred training approaches, and implementation considerations within rural Ugandan communities.

### Strengths and limitations

This study has several limitations. It was conducted in a single rural district in southwestern Uganda, which may limit the transferability to other regions with different cultural, socioeconomic, or healthcare contexts. Participants were recruited through a community-based organization providing services to older adults. Their experiences and training priorities may differ from those of caregivers who are not connected to organized support services. The qualitative design and small sample size may not capture the full diversity of caregiving experiences nationwide. Additionally, only three key informants were interviewed, and therefore healthcare provider perspectives may not fully represent the range of professional views regarding dementia care in Uganda.

Despite these limitations, the study has notable strengths. It provides in-depth insights into caregiver-defined training needs in a rural Ugandan context, a population that remains underrepresented in dementia research. Inclusion of both informal caregivers and key informants enhanced credibility through triangulation. By focusing on caregiver-identified priorities, this study offers actionable evidence to inform intervention adaptation rather than solely describing caregiving burden.

## Conclusion

This study highlights the complex challenges faced by informal caregivers of persons with dementia in rural Uganda, including limited knowledge of dementia, substantial caregiving burden, and unmet training needs. The findings suggest that caregivers would benefit from targeted culturally appropriate caregiver training interventions focusing on communication, management of dementia-related behaviors, personal hygiene, nutrition, and self-care. Addressing misconceptions about dementia through community sensitization, alongside caregiver training, may foster more supportive care environments. These approaches have the potential to improve caregiving experiences and the quality of dementia care in rural, low-resource settings.

## Supporting information

10.1017/gmh.2026.10279.sm001 Namwase et al. supplementary material 1 Namwase et al. supplementary material

10.1017/gmh.2026.10279.sm002 Namwase et al. supplementary material 2 Namwase et al. supplementary material

10.1017/gmh.2026.10279.sm003 Namwase et al. supplementary material 3 Namwase et al. supplementary material

## Data Availability

The original contributions presented in the study are included in the article/supplementary Material, further inquiries can be addressed to the corresponding author.

## List of abbreviations

LMICs: low- and middle-income countries
ROTOM: Reach One Touch One Ministries
WHO: World Health Organization

## Supplementary material

The supplementary material for this article can be found at http://doi.org/10.1017/gmh.2026.10279.

## Long descriptions

### Table 1. Long description

The table contains six columns: Participant, Sex, Age in years, Highest level of education, Relationship to person with dementia, and Duration of giving care.

* P 1: Female, 40, Primary, Mother, 10 years.

* P 2: Female, 48, Primary, Mother, 3 years.

* P 3: Female, 54, No formal education, Father-in-law, 6 years.

* P 4: Female, 25, Primary, Grand parent, 5 years.

* P 5: Male, 75, No formal education, Wife, 8 years.

* P 6: Female, 62, Primary, Mother, 2 years.

* P 7: Male, 42, Primary, Grand mother, 7 years.

* P 8: Female, 40, Secondary, Mother-in-law, 6 years.

* P 9: Female, 50, No formal education, Mother, 5 years.

* P 10: Female, 66, Primary, Sister, 13 years.

* P 11: Female, 60, Primary, Mother, 9 years.

* P 12: Female, 58, Primary, Mother-in-law, 3 years.

* P 13: Female, 60, Primary, Mother, 13 years.

* P 14: Male, 36, No formal education, Mother, 24 years.

* P 15: Female, 75, No formal education, Husband, 22 years.

* P 16: Male, 30, Secondary, Not related, 7 years.

* P 17: Female, 58, Primary, Father, 30 years.

Navigate back to Table 1..

### Table 2. Long description

The table consists of five columns: Participant, Gender, Age in years, Profession, and Years of experience.

* Key informant 1 is a 30 year old female Nurse with 11 years of experience.

* Key informant 2 is a 28 year old female Clinical officer with 1 year of experience.

* Key informant 3 is a 31 year old male Medical officer with 4 years of experience.

Navigate back to Table 2..
