# Supplementary material for: Training needs of informal caregivers of persons with dementia in rural southwestern Uganda: A qualitative formative study
Source: Glob Ment Health (Camb). 2026 Jul 30;13:e171. doi: 10.1017/gmh.2026.10279 (PMC13458857; doi:10.1017/gmh.2026.10279)
Supplement: Supplementary file 1 [file S2054425126102799sup001.pdf]

INTERVIEW GUIDE FOR INFORMAL CAREGIVERS

Date of Interview: …………………… Venue of Interview: …………………… Interview starting time: ………… Interview ending time...

Respondent ID: ....

Socio-demographic information

I. Age……………………….

II. Sex ………………………….

III. Highest level of education: ……………………

IV.Current role in the community………………………………………..

V. Relationship to care receiver? ……..………………..

VI. How long has the patient been living with dementia………………

VII. How long have you cared for the person?.…………………..

VIII. Housing Status: a). Rented b). Owned (permanent)

c). Temporary shelter

IX. Household income level ………………….

Caregiver background and experiences

1. What do you know about dementia? (general understanding, knowledge about symptoms, disease progression, care?)
2. What do you think causes dementia? (Probe for reasons they think that way)
3. What do you think happens to people with dementia as the disease progresses? (probe for signs of trouble remembering, misplacing things, communication etc)
4. What do you do if you think that your loved one has dementia? (Probe for what they have done)

Challenges and coping strategies

5. Please tell me about your typical day as a caregiver of someone with dementia or signs of dementia
6. How have your roles changed over time while caring for someone with dementia or signs of dementia?
7. How do you stay connected with the person with dementia or signs of dementia? (probe for communication, talking to other people about your patient)

8. How do you relax away from caretaking the person with dementia or suspected dementia? (Probe for ways of reducing stress, making time for pleasant activities, staying in control of own feelings)
9. How have you involved others in care (e.g., friends, family members?
10. What do you think about the future of a person with dementia?
11. How do they feel about having a family member bathe them?
12. What does the person care for want to do with themselves as long as possible?
13. If more assistance is needed at home, what kind of help would the person you care for prefer?
14. What are their wishes at the end of life (if at all they can speak about it)?

Training related questions

15. What do you think about receiving training in caregiving for people with dementia or suspected dementia? (probe for willingness to attend, possible barriers to attendance)
16. What information would you like to receive to improve your caregiving role?
17. What would be the most appropriate format of delivery? (In-person training, educational materials)

KEY INFORMANT INTERVIEW GUIDE

Date of Interview: …………………… Venue of Interview: …………………… Interview starting time: ………… Interview ending time... (45-60) minutes

Participant ID: ....

Demographic information

I. Age (years)

II. Sex ………………………….

III. Level of education/ qualification ……………………

IV. Designation ....………………………………………….

V. Duration working with Dementia Caregivers………………

Understanding caregiver challenge

1. From your experience, what are the most common challenges informal caregivers face while caring for people with dementia? Probes: Diet, Stress, Sleep Disturbance, Making time for pleasant activities,

2. What are the most common questions or concerns that caregivers bring to you when seeking guidance on dementia care?

3. Are there specific areas where you see caregivers struggling most (e.g nutrition, hygiene, sleep)?

Caregiver knowledge gaps

4. In your opinion, what critical information do caregivers lack when caring for people with dementia?
5. Are there any misconceptions about dementia care that you frequently encounter from caregivers?

Caregiver skills and abilities

6. What practical skills do you think caregivers need to improve in order to provide better care for dementia (e.g. medication, hygiene, agitation)?

Emotional and psychological support for caregivers

7. What kind of emotional / psychological support do you think caregivers need?
8. What kind of emotional / psychological support do you think are caregivers currently receiving?

Training needs of caregivers

9. What specific areas of dementia care do you believe caregivers need more training? (e,g communication)
10. If there were a training program for caregivers, what topics should be prioritized to improve their caregiving skills?
11. How do you think cultural beliefs and practices impact caregivers' willingness or ability to adopt new caregiving techniques? Should these be considered in the training?

Preferred training methods for caregivers

12. From your perspective, what are the most effective ways to deliver training to informal caregivers e.g. workshop, one on one, online, in person?
13. What would you like to be (topics or content) in a training program for caregivers like yourself?
14. What kind of resources would be more beneficial e.g online, hard copy? How would you prefer to receive this training? (Probes: workshops, one-on-one, written materials)
15. How can training be organized to accommodate those with limited time and access to resources?

Resources and support for caregivers

16. What resources do you provide to caregivers? (probes are they sufficient)
17. How can H/W better support these caregivers in accessing these resources or caring for people with dementia?
18. What additional resources (e,g hotlines, pamphlet) would be helpful?
19. Anything else to share on this topic?
