# Supplementary material for: Training needs of informal caregivers of persons with dementia in rural southwestern Uganda: A qualitative formative study
Source: Glob Ment Health (Camb). 2026 Jul 30;13:e171. doi: 10.1017/gmh.2026.10279 (PMC13458857; doi:10.1017/gmh.2026.10279)
Supplement: Supplementary file 2 [file S2054425126102799sup002.pdf]

Supplementary File 1: COREQ 32-Item Checklist

Title: Training Needs of Informal Caregivers of Persons with Dementia in Rural Southwestern Uganda: A Qualitative Formative Study

Authors: Joyce Namwase, Julius Kyomya, Amos Oyuru, Emmanuel Maleka, Abraham Muhwezi, John Semuwemba, Christine K. Karungi, Celestino Obua, Edith K. Wakida

No.	Item	Guide question	Response	Section / Page
**Domain 1: Research team and reflexivity**				
**Personal characteristics**				
1	Interviewer/facilitator	Which author/s conducted the interviews or focus groups?	Trained research assistants Jenipher Asasira and Patricia Tushemereirwe	Methods: Data collection tools on page 7
2	Credentials	What were the interviewers' credentials?	Both held Bachelor's degrees in Social Science	Methods: Data collection tools on page 7
3	Occupation	What was their occupation at the time of study?	Research assistants in the study team	Methods: Data collection tools on page 7
4	Gender	Was the gender of the interviewers reported?	Female	Methods: Data collection tools on page 7
5	Experience and training	What experience or training did the interviewers have?	Both had prior experience conducting qualitative research and were trained in in-depth interviews for this study	Methods: Data collection tools on page 7
**Relationship with participants**				
6	Relationship established	Was a relationship established prior to study commencement?	There was no prior relationship with participants but rapport was established by scheduling of interviews and at the beginning of interviews	Methods: Ethics approval and consent to participate on page 8

**7**	Participant knowledge of the interviewer	What did participants know about the interviewer?	Interviewers introduced themselves as trained research assistants conducting a study on dementia caregiving.	Methods: Data collection tools on page 7
8	Interviewer characteristics	What characteristics (bias, assumptions, reasons) might influence the study?	Interviewers were trained to be neutral, empathetic, and culturally sensitive and had prior qualitative research experience.	Methods: Data collection tools on page 7
**Domain 2: Study design**				
**Theoretical framework**				
9	Methodological orientation	What theoretical framework or approach?	The study used qualitative descriptive approach with thematic analysis	Methods: Data management and analysis on page 7
**Participant selection**				
10	Sampling	How were participants selected?	Purposive sampling based on caregiving role and variation	Methods: Study population, sample size, and sampling on page 6
11	Method of approach	How were participants approached?	Participants were contacted via ROTOM registry and invited by phone calls.	Methods: Study population, sample size, and sampling on page 6
12	Sample size	How many participants?	We had 17 caregivers, 3 key informants	Methods: Study population, sample size, and sampling on page 6
13	Non-participation	How many refused or dropped out?	None refused or dropped out.	Methods: Data collection tools on page 7
**Setting**				
14	Setting of data collection	Where were interviews conducted?	Interviews were conducted at participants' homes or workplaces	Methods: Data collection tools on page 7

15	Presence of non-participants	Was anyone else present?	No one was present besides the research assistant	Methods: Data collection tools on page 7
16	Description of sample	Characteristics reported?	There were 15 female and 5 male participants	Results: Participant characteristics on page 9
**Data collection**				
17	Interview guide	Were questions/pilots provided?	Participants were presented with prompt open ended questions starting anywhere they felt comfortable but completed unstructured interview. It was not piloted. The questions were translated into Runyankore-Rukiga	Methods: Data collection tools on page 7
18	Repeat interviews	Were repeat interviews conducted?	No repeat interviews were conducted	Methods: Data collection tools on page 7
19	Audio/visual recording	Were interviews recorded?	Interviews were audio-recorded with consent	Methods: Data collection tools on page 7
20	Field notes	Were field notes taken?	The research assistants took field notes	Methods: Data collection tools on page 7
21	Duration	Length of interviews?	The interview lasted between 30 to 60 minutes	Methods: Data collection tools on page 7
22	Data saturation	Was data saturation discussed?	The sample size was sufficient for saturation	Methods: Study population, sample size, and sampling on page 6
23	Transcripts returned	Were transcripts returned to participants?	Transcripts were not returned to participants.	Methods: Data management and analysis on page 7
**Domain 3: Analysis and findings**				
**Data Analysis**				

24	Number of coders	How many coders?	There were 6 coders JN, JK, EM, AM, AO and JS	Methods: Data management and analysis on page 7
25	Description of coding tree	Were themes/coding tree described?	The themes were manually developed and iteratively refined	Methods: Data management and analysis on page 7
26	Derivation of themes	Were themes pre-defined or emergent?	The themes were derived from data (inductive)	Methods: Data management and analysis on page 7
27	Software	Was software used?	Manual coding was done without any software	Methods: Data management and analysis on page 7
28	Participant checking	Did participants provide feedback on findings?	No feedback was provided by participants	Methods: Data management and analysis on page 7
**Reporting**				
29	Quotations presented	Were participant quotations presented?	The quotes were presented with participant number, gender, and age	Results: Themes on page 10
30	Data and findings consistent	Was there consistency between data and findings?	There is consistency between data presented and the findings.	Results: Discussion on page 14
31	Clarity of major themes	Were major themes clearly presented?	Five main themes were presented	Results: Themes on page 10
32	Clarity of minor themes	Were minor themes described?	Subthemes were included under each major theme	Results: Themes on page 10
