# Supplementary material for: Training needs of informal caregivers of persons with dementia in rural southwestern Uganda: A qualitative formative study
Source: Glob Ment Health (Camb). 2026 Jul 30;13:e171. doi: 10.1017/gmh.2026.10279 (PMC13458857; doi:10.1017/gmh.2026.10279)
Supplement: Supplementary file 3 [file S2054425126102799sup003.pdf]

Informal caregivers

**Main domain**	**Thematic area**	**Subtheme**	**Quote**
**Caregiver experience**	Knowledge about dementia	Understanding about dementia	"*I don't know but she suddenly changed, she would place something somewhere and forget and I would wonder what the cause was but later thought it was old age. Then sometimes she would remain like that but remained forgetful. Later we decided to leave her alone, when she would put something somewhere, we would remove it and keep it but she would wonder where it is and look for it but fail to find it*."<br>[At the facility] "*They said when people grow old, they became like that and advised us not to annoy them and also not to be rude if they asked about something.*" **Caregiver 1**<br>*"When you tell her something, she forgets after sometime or when I put for her something to drink and inform her, later when you go to check you find she has forgotten to take it but we do not know the cause of this."* **Caregiver 2**<br>"*There are times when you overthink and you appear like you are getting some mental disorientation, so once it sets in; you find yourself misplacing things, for example, you may place a knife there and look for it everywhere even while by passing it in the process or you look for something which you have in your hands and this happens as a result of overthinking. It may also come about as a result of an accident. For example, if someone gets an accident, they may end up getting mental challenges.*" **Caregiver 3**<br>"*We were there and she just started forgetting and I asked her why it was so and she said she had no idea why. She would place something somewhere and later on she would start asking me where she could have*

			placed it. I would ask when she gave it to me but she wouldn't have an idea, it would be the beginning of a quarrel. Slowly by slowly it progressed. She would prepare food and no water and it would get burnt. How does one prepare food and fire starts coming out of sauce pan before noticing that there is no water? I told her, 'from today always leave the food I can always prepare food for myself because you can no longer manage." **Caregiver 5**<br>"*Factors that can contribute to dementia may include experiences perceived as spiritual attachments or disturbances, which individuals often associate with their family circumstances. Because when we started praying, she became better and, in most cases, when mad people are taken to churches for prayers they get better.*" **Caregiver 6**<br>*It may be attributed to her old age, because for someone who is 94 years, they are really old, so I think it is the reason she got Dementia –* ***Caregiver 7***<br>*This condition confuses me because you can be discussing with someone, and within a minute, they have already forgotten what you were talking about.* **Caregiver 8**<br>*He forgets, he gets mental deterioration, even when it comes to talking, the information does not come out well* **caregiver 17**<br>*When she got dementia, I realized that she got mental disorientation, to an extent that she cannot understand what you are telling her. She starts telling you things that are not coordinated or even connected to what you are asking her* **Caregiver 14**<br>*What I know about it, he really forgets, even when it comes to communication, he talks of things that are not connected, at times when you are there, he can be like, "The goats are spoiling people's gardens",*

			and yet he is looking at the curtain in his room. So that is the most challenging thing I have seen with it, he also talks to himself. **caregiver 16**<br>*In the past she was ok and bright, but now she has some mental disorientation. For example, when you serve her food in a dish, she claims it that it's hers. She can ask that how did this reach here.* **caregiver 12**<br>*I don't know it, maybe I also have dementia, because even me, on many occasions I tend to forget, like sometimes when I have entered the house looking for something, and when I reach there, I forget, then I end up going back to pick it, so I don't know what causes this kind of forgetfulness in a person.* **caregiver 13**<br>*I used to hear it among other people, until it finally affected my own mother, for example, she forgets. Sometimes you can go there and hear her say, "The house is on fire", she gets properties and starts throwing them out of the house, we get ropes and tie her up. She usually gets a mental disorder, so we take her to the hospital or take her for prayers, and she gets better, after sometime she again gets sick, sometimes when they pray for her she starts chewing herself as if she has something in her mouth that she is chewing* **caregiver 11**
		Causes of dementia	*"I think excess worrying and stress because before we were born, we had elder siblings that grew up when they were going through a lot of hardships so I think that when she dwells on such matters, she loses her sanity."* ***caregiver 1***<br>*"When she got very sick, I assumed that that's where the forgetfulness came from. Because she fell sick for four years and she lost her sense of*

			speech and she became deaf. That is where we think the condition originated.<br>*I think it is because she was severely sick, then also the strenuous work she used to do that she failed to continue doing so I think she got stressed leading to dementia." **Caregiver 2***<br>*"I failed to understand this disease, I failed to know it (perplexed), no what do I say caused it? Even us we are growing old and I see such dementia developing I wonder what causes it, so I can't lie to you I don't know what brings it." **Caregiver 5***<br>*I think it is due to old age **Caregiver 9***<br>*"I think, just as someone can be born with a physical disability, her disability became dementia. Even when she was young, you could send her for something, and she would bring the wrong thing, and the situation gradually worsened. She wouldn't have been able to manage marriage because if someone can't cook, they can't manage a husband." **caregiver 10***<br>*"If I were to develop dementia now, I would attribute it to old age. We are advised to eat foods that boost brain function, but due to poverty, we can't always afford them. …" **caregiver 10***<br>*"Another cause of dementia can be the overuse of medications, such as those for high blood pressure or diabetes, which can weaken the body as time goes on. Since these medications are taken daily, they can gradually affect brain health, making the condition worse." **caregiver 10***<br>*"Its madness, since she has ever run mad. In fact, we were worried that maybe she had HIV because in most cases HIV patients get mental disorientation" **caregiver 6***<br>*other illnesses also attribute, diabetes is a bad disease **caregiver 17***

			I was thinking maybe in family, they worship the devil and probably they initiated him to the devil, that was my thinking. But they said that it is due to old age ***Caregiver 15***<br>*……… after my father died, she had a mental breakdown and ran mad. I took her to traditional healers, and she recovered, but she continued to develop other illnesses. I think the other reason could be her desire for me to get a wife. I got married and separated with my wife after our first child, and it bothers her so much. There is a time she was sick when she got better, she told me, "My child, why don't you get a wife and settle, do you want me to die and leave you with nothing*." **caregiver 14**<br>One is high blood pressure, because when he thinks of his personal problems, they add on disturbing his mind. Secondly, the other stroke that he got twice, there is a way it also affected his mental understanding, so I think those are the causes **caregiver 16**<br>I think it is because she over thinks, because she consecutively lost all of her first three children, then later she lost her last born, making them four**. caregiver 13**<br>I think it is because she lost her children. Do you know when you lose your children you get mental disorientation? **caregiver 12**<br>usually tell us, that it is due to old age, and the rest of the explanation is always in English. **caregiver 11**
		Signs and symptoms of dementia	"*Nowadays she forgets a lot. You may be sited with her talking and after a few minutes she asks the children whether I have already come home yet I am seated next to her. So I realized that even when I am with her, her mind is not present with me.*" **Caregiver 1**

			"*Now, the symptoms that show signs of severe forgetfulness. You first observe it in the things he does. Okay you notice a difference in their ways, for example, you can give him something or when you ask him something; and after a while he responds to your questions, at times telling you uncoordinated things. So that is how you notice the signs of mental disorientation are progressing slowly and with time, it keeps growing*." **Caregiver 3**<br>"*Those are the common signs of people with that condition. They tend to forget a lot for example, I may place here a knife, and look for it all over and even borrow another knife from elsewhere while bypassing it*." **Caregiver 3**<br>She would place something somewhere and forget and go away then realize she has left something behind and return to pick it. **Caregiver 4**<br>"*When going to urinate, she has no control over it. When going to pass stool if she doesn't move fast then she will soil herself so I think such symptoms are also there as a cause of dementia*." **Caregiver 4**<br>"*We were there and she just started forgetting and I asked her why it was so and she said she had no idea why. She would place something somewhere and later on she would start asking me where she could have placed it. I would ask when she gave it to me but she wouldn't have an idea, it would be the beginning of a quarrel. Slowly by slowly it progressed. She would prepare food and no water and it would get burnt. How does one prepare food and fire starts coming out of sauce pan before noticing that there is no water? I told her, 'from today always leave the food I can always prepare food for myself because you can no longer manage*." **Caregiver 5**

			*She is the one who told me, she would say, "I cannot recognize some people, my eyes no longer see well"…**Caregiver 7***<br>*The time she was staying alone in the house, she'd keep things and forget where she has kept them, for example, there are times when she would be like, "I came with something, but I don't remember where I put them". So, I thought maybe it was due to old age,...**Caregiver 7***<br>*Misplacing things, trouble looking for them and yet they're just besides her. There you can know that forgetting has set in.She can be going somewhere but she ends up forgetting where she is going.* **Caregiver 8**<br>*She got a hearing problem. When she stopped doing things for herself, for example, digging, we realized that she was not ok **Caregiver 9***<br>*She used to throw things out of the house, like knives, she would misplace things, we look for them and we wouldn't find them.* **caregiver 6**<br>*Its running away and forgetting his children**,*** **Caregiver 15**<br>*talks about people who died long ago, those in the past, and those he has spent long without seeing* **Caregiver 15**<br>*Talking to himself, you may find there is no one else in the room but he is talking to himself* **caregiver 16**<br>*when you tell her something now, tomorrow if you ask her about it, she will ask you that when did they talk about it. She can be like, "When did they talk about that?" And then I try to remind her how we were discussing about it the previous day**.*** **caregiver 13**<br>*Now like this tank that was donated by ROTOM, my husband, children, and I are the ones who contributed the bricks and sand to build where it is located, but she does not allow us to fetch water from it* **caregiver 12**

			She cannot remember where she places her things, for example, she misplaces them to an extent of telling you that maybe they are at your place yet they are all in her house **caregiver 12**<br>*For her she does weird things, when she gets a mental problem, for example, you can be there and she tells you, "I have gone to "Kabale", and you find her on the way, where you passed when you are coming. In fact, last week she dressed up, got a boda boda and left, they called me to pick her from Bukyinda" in someone's home, I got a boda boda, went and picked her up. So, you may think that she is normal, but she is not she is on and off, she may be normal and ok now, but in a few hours to come, she changes and starts doing weird things* **caregiver 11**
		Progression of dementia	"*Before she used forget but later remember where she has placed something but now she no longer remembers anything. Nowadays we are very mindful, if you place something somewhere for her, you have to again pick it and personally hand it back to her. If you leave it say food, she will tell you when you return that she has not yet eaten when we have already served her. So we have to come and hand her the food. Let's say I tell her to wake up but she responds that it's not yet dawn yet she is hearing that we are already outside.*" **caregiver 1**<br>"*They develop habits, become grumpy and irritable. You notice some of that behavior like when you put some food on a plate and she says she doesn't want that particular plate and the one she wants at times we don't have it. So when you put on that plate she will say she doesn't want the food but later say they have refused to give me food and eventually she fails to get what she wants*."

			**"***She no longer has control over her bladder as before, by the time she sits on the container to urinate, the urine would have spilled and she also falls or trips so often. That is how she is.* **"caregiver 1**<br>"*I think the disease will deteriorate because her years will be increasing. Even she won't be remembering the little she does now*." **Caregiver 1**<br>"*She doesn't express herself the way she used to. I don't know whether it's because she doesn't hear well so like when I am conversing with you and we laugh, she assumes that we are backbiting her. Then she gets annoyed but then we calm her and tell her that we were not talking about her then she cools down*." **Caregiver 2**<br>**"***When you are staying with someone, it is easy to notice a difference. There are times when he becomes argumentative, so when you respond and both of you end up quarrelling. So, in that case you have to ignore him because you know that he is mentally disoriented. And in most cases when you ignore him the situation stabilizes, though he is on and off because he still argues sometimes***." caregiver 3**<br>"*By the time one always misplaces things or forgets the previous location of items. It's hard to believe that this forgetful tendency will elapse. I think the more you grow, the more the disease progresses because as one ages, the memory keeps failing.*"<br>**"**The way I think it will become hard on him as he grows older, his intellect and reasoning capabilities will keep diminishing, and as this goes on, he will not ponder on the past so that he can control his ambiguity the so that the condition can subside. This disease will keep progressing with his age.**" Caregiver 3**

			"*She places an item somewhere after some time she looks for it and asks where it was. Sometimes she forgets where she has placed it. When you ask her where she put something she can't remember."* **Caregiver 4**<br>*She cannot do anything for herself. One has to bathe her, apply Vaseline, waking up is difficult and she is always complaining when getting up.*<br>**Caregiver 4**<br>"*If it progresses we are going to suffer. She is like a baby, you see her soiling herself, urinating on herself and also touch faeces like a baby. Now she has reached a point of walking naked what do you think is the next step. Now if I am not caring for her she can even walk to the Centre while naked. One day I met her going to the trading centre naked. For her she is unaware of anything.*<br>*You will find her changed, will be like a child. She has lost wisdom at this point and yet the disease progressing, so won't she do childish things? She will do childish things, defecate and eat fecess like children*."<br>**Caregiver 5**<br>"*As it progresses I believe this is a result of what is referred to as "Ebyamaka," which translates to "ancestral spirits." Such influences can harmfully affect the family, potentially leading to mental health issues among its members including running mad."*<br>*The future for those with dementia can involve challenges such as defecating and urinating in one place, as they become mentally disoriented. It's important to understand that when caring for an elderly woman, it may be necessary to create a hole in the mattress so that they keep urinating through that hole. When someone reaches old age, he/she becomes like a little child, making it difficult to carry them all the time*."<br>**Caregiver 6**

			*Yes, that is it they misplace things, they even forget the things they had been thinking about, even when you are talking to them, you realize that she has changed, she keeps repeating whatever they have already said…**Caregiver 7***<br>*I think this person can end up being mad or running mad. She has been doing all that, when you ask her something, she starts telling you things that are not connected to the question, she tells you things of the past, like how she was well in the past and how things have changed now.* **Caregiver 8**<br>*It is you to take care of that person as they are not able.* ***Caregiver 9***<br>*those days he used to walk, he was working, but now he cannot do anything, everything has totally failed* **Caregiver 17.**<br>*We bathe him, I have to hire people to help me to carry him. I boil water and take it to the bathroom, we bathe him and then carry him back to his bed* **Caregiver 17**<br>*When they come and introduce themselves, he recognizes them from their voices, but when you just come, he may not recognize you, because he does not see, in other words if your voice is not familiar, he cannot recognize you* **Caregiver 17**<br>*The time I came, he could talk to someone and they understand him, but it was on and off. At times he would talk of things that are not connected, and then other times he would talk normally, and we understand him. But due to other conditions like high blood pressure and diabetes, he also got stroke* **caregiver 16**<br>He uses a catheter **caregiver 16**<br>*Not really, except that even walking has failed, he has developed very bad bed sores.* **Caregiver 16**

			……….. So, I think outcome for dementia is death, because there is no cure for it. **caregiver 16**<br>Yes, she wets the bed, in fact the ROTOM people gave her a Mackintosh which we use. She is like a little child. I think if she gets drugs she's likely to improve and be better, but if she fails to get it the mental disorientation may increase until she dies **caregiver12**<br>*For example, before he got to this condition, he would give himself something to drink, he even used to wash for himself, but now because of old age, he no longer can be able to wash, I have to come in and help* **Caregiver 15**<br>In my opinion, I think as she grows, even the disease progresses, because every day her health deteriorates, and I just keep praying that God takes care of her. Usually when the situation becomes worse, I take her to ROTOM, Muhanga, for treatment. In fact, I last took her there in November last year **caregiver 14**<br>At first, we used to tie her with ropes, and lock her up in a room, we would serve her food from there, and give her what to use in case she wants to ease herself. I would give her the medication, after like two days, she gets better, after some time, she starts chewing herself, as if she is chewing something, then you would also find her talking of things that are not adding up.**caregiver 11**<br><br>*Yes, because like in the previous days, she used to wake up early like at around 6am, but these days, if I don't wake her up, sometimes she wakes up at around 9am or even 10am, that means that she keeps getting weak and weaker. Usually, when you have the strength, when it clocks 6am,*

			you feel like leaving the bed, but if you find yourself getting weak and weaker that means the disease is also progressing **caregiver 11**
		Care/ treatment needed	"*It progresses so we close the door and leave her in the room so that if she disorganizes this place we can always put the things back in order when we return*." **Caregiver 1**<br>*"It* [the medication] *helped a bit but the forgetfulness still returns after some time*." **Caregiver 1**<br>"*We had not yet thought of taking him to the hospital specifically to treat dementia, because we thought it was due to old age and the accident, he got that causes his forgetfulness.*" **Caregiver 3**<br>"*I felt bad about it, but I had no choice because there is a hospital that takes care of people like him, when he falls sick, he goes for treatment. But we had not taken him specifically to get dementia treatment."* **caregiver 3**<br>*"The way I look after this old lady, I try to look for pumpkin seeds, greens because they said those would reduce forgetfulness. I prepare them for her. There are times when she isn't very forgetful so that's why I feed her on that."* **Caregiver 4**<br>"*You take him/her to the hospital for treatment and if the hospital fails to help, you take them for prayers every night may be prayers can work*. **caregiver 6**<br>"*When we took her to Rugarama hospital to rule out our suspicions because we thought of many things including cerebral malaria, and there was no improvement, I sought advice from a friend who advised me that whenever she leaves this place, I should not follow her, instead I should get a cross (that one you have seen) and pray for her that, "God you are*

			the one who has taken your child, may you be the one to bring her back". So, whenever I would pray, she would come back and tell me that, "God told me to come back home and never to spend a night in the forest," and indeed, from that time she has never spent a night in the forest again, I was excited." **caregiver 6**<br>*Yes, it is not a disease, it is just mere forgetting just misplacing things* **Caregiver 9**<br>"*When caring for a dementia patient, it is important to keep loving them and forgiving their mistakes because they are not themselves. For instance, my sister sometimes goes out onto the road and starts stopping vehicles when we are not around. When drivers ask her where she is going, she simply says she was just stopping the vehicles*." **Caregiver 10**<br>*You must be patient with her, especially if she keeps on repeating things or repeating the same thing over and over due to old age, I believe that's not a disease that requires medical attention* - **Caregiver 7**<br>*We took her to the hospital because ROTOM is there, they give her treatment, once she gets better, and she comes home. So, every time she's not ok, we take her there for treatment, when she gets well, we bring her back home. There are times we have even taken her to Kampala. She has a son in Kampala, so at times he asks us to take her there. In fact, he is the one constructing that house over there. But ROTOM has helped her so much, otherwise, she would not still be alive. Someone of her age cannot spend a week without going to the hospital* – **Caregiver 7**<br>*We took him to several hospitals to seek treatment including; Kampala, Mbarara, Kabale and ROTOM. Because it is near, when I fail to manage him at home, I bring him here* **Caregiver 17**

			I would rush to hospital so that i can get counselling and guidance from the hospital **Caregiver 14**<br>*We took him to the hospital in Muhanga for treatment, he was admitted for one and a half weeks, when he got better, we took him home.* **Caregiver 15**<br>*Yes, they should all be taken to the hospital because that is the only place to get help, it's a health garage* **caregiver 16**<br>*I did not take it as a disease or an illness, I just thought it is the usual forgetfulness* **caregiver 13**<br>You asked me if we have ever taken her to the hospital to get medical attention. Is there any medication for dementia?<br>*We took her to ROTOM for treatment, and they even gave her medicine for putting in her ears but there is no improvement* **caregiver 12**<br>*We rushed her to the hospital at ROTOM in Muhanga, they carried out several tests including, blood pressure, diabetes* **caregiver 11**
		Prevention of dementia	
**Challenges and coping strategies**	Routine	Caregiving tasks	"*In the morning, I cook food or prepare black tea and she takes it, then I go to dig, at lunch time I come back and prepare lunch and give her*." **Caregiver 1**<br>*"At times when I wake up I sometimes want to prepare for her porridge but I don't have flour or sugar so I just boil water and give her. Then, I go to dig and when I return, I prepare food and we eat.*" **Caregiver 1**

			“*I bathe her, wash her clothes and she stays inside. I lock so she only goes to see the goats and returns to the house so we find her when we return. .. I bathe her at midday because she cannot bathe cold water in the morning. Then there are times when I warm the water in the morning and she says she doesn’t want to bathe so I usually do the bathing when the sun is hot.*” **caregiver 1**<br>“*I usually put water in a basin and a stool and she bathes but nowadays I have to help bathe her and scrub her back.*” **Caregiver 1**<br>“*When I wake up, I prepare breakfast when I have some maize flour and call him to take with his brother. We later prepare lunch and they come and eat, supper is the only meal that we serve him in his house but he eats usually lunch and breakfast with his brother*.” **Caregiver 3**<br>“*So I make sure I wash her clothes very well. She sits close to me on a bench to ensure I have used clean water to rinse, when water used is clear then she will know they are clean.”* **Caregiver 2**<br>“*I wake up and give her medication, after I prepare breakfast. She usually wakes up at 9 am since she isn’t strong enough. Then I remove beddings for washing and relay her bed. I give her the breakfast and start on lunch, after I go to dig and then bring her a drink as I prepare supper. In the evening, I lay her bed and she enters bed and sleep*.” **Caregiver 4**<br>“*I also wash her clothes when they get dirty because I cannot give her tasks she can’t manage. I treat her gently, and when she is happy, she plays for us and makes us laugh, she is fun to be with during those moments. However, her mental disorientation affects her eating, if you interrupt her to tell her something, she forgets that she was eating and doesn’t go back to finish her food, leaving it for the hens instead. Dementia is truly a challenging condition..*” **Caregiver 10**

			“*When I wake up in the morning, the first thing I do is check her bed to see if she soiled it, then I greet her to see how she responds and prepare her breakfast. I can’t take her to the garden because I know she won’t be able to do much. As I mentioned before, she spoils the beans instead of weeding, and in the Irish potato garden, she pulls up the plants and throws them away, even though they aren't ready for harvest.*<br>*So, I just make sure she has something to drink, and I try to be gentle with her. Sometimes, when I leave her in charge of something, it gets messed up, but I know it’s because she forgets. She requires a lot of patience, and I always remind myself of that*.” **caregiver 10**<br>“*Now there are times when I see that she is not clean, I warm water and I take to the bath room and bathe her, “ have you understood me?” Even clothing also when they become dirty I wash them and even when I see her hair not looking nice I also cut it off, “have you heard?” That is how I care for her.*<br>*For her she wants to be naked, one day we pulled each other when she woke up wanting to go outside naked and I told her stop, what will people who see you naked think, I put her back in the bedroom, and forcefully dressed her, she removes it I put it back.*<br>*She is like a baby, do you think she knows how to bathe, sometimes I put water on the basin and tell her to bathe but she pours the water by overturning the basin, she is like baby, she doesn’t understand anything*.” **Caregiver 5**<br>“*I wake up like this, I start by sweeping her home and doing any necessary cleaning. If her bed is soiled, I clean it and wash the bed sheets. She enjoys cold drinks, so I prepare something for her and let it*

			cool. For lunch, I make her favourite foods, including greens, sorghum porridge, and posho, and then serve her.” **caregiver 6**<br>*Yes, I am the one who brings her milk, bread, sugar and meat to make her happy. And she still walks normally, she still can be able to walk and she can even take herself to Muhanga Town, and even take herself to the garden, she even cooks. –* ***Caregiver 7***<br>*She has been cooking for herself, even when the husband was still alive, but when he died, we realized she could not stay in the house alone, and therefore decided to bring her a maid so that she gets someone to give her company. But she has been staying here with her husband –* ***Caregiver 7***<br>*When I wake up in the morning, I first take measurements for blood pressure and diabetes and record the readings. After that, I brush his teeth, give him a bed bath, change his clothes and beddings, give him breakfast, after breakfast I take him out for sun bathing, after sunbathing, I take him back to the house and he sits in a comfortable chair, he leaves the chair when he is going back to bed, at around 1pm, I serve him lunch, I keep monitoring him until supper is ready or if it is still shining in the evening, I again take him out for sun bathing, we leave outside when he is going for supper and the day ends like that****. caregiver 16***<br>*The good thing with my mother, she lays her bed by herself, whichever way she does, except when she is sick and badly off to an extent of carrying her in and out of the house. That’s when I help her, it’s because she doesn’t have the energy to do it herself or I also know I have to help in tidying the basin where she urinates at night. I get her some water to washe some parts of her body. Then I help to get her something to eat and she does not disturb me because she doesn’t have good appetite.*

			She is selective, and the doctor advised that we should always get her soft foods like yellow bananas because she has chronic constipation. So, after getting her a warm drink, she takes her two yellow bananas and when you get her some food, she prefers to take it on time ***caregiver 13***<br>*When you wake up, you first check on her and greet her, ask and find out how her night was, then you take her out to sunbathe if at all the sun is there, if there is breakfast, you serve her, you give her water for bathing and go to do other things. Sometimes I also bathe her but it makes her uncomfortable.* **Caregiver 8**<br>*I wake up early, I first greet him or check on him, to see how he slept, then I pray for him. I boil water and bathe him, I prepare for him breakfast, although he doesn't drink much because of little strength, I make sure that he takes something, at least like water and may be little food. So, after that, he stays in bed, and I go to prepare lunch, then I force him to eat because it is difficult for food to pass on the throat. In the evening, I prepare for him some tea. Of course, along the day, I have to do Washing, so there is a rest. I have a lot to do, there are times when I do not have a house help, you cannot have one every day, so during that time, it is always a difficult moment, I get tired, I suffer, but I have to persevere, I have no choice*. **Caregiver 17**<br>*The ROTOM people instructed me to always prepare greens for him every day. Then to prepare two cups of water to give him every day, in fact before I left home, I told the lady at home, to give him that water, I'm sure by the time I get there, I will find when he has already taken.* **Caregiver 15**

			When I wake up in the morning, I check on her, wake her up, and remind her to clean up. Then I prepare her breakfast, which might include milk, sugar, buns, and shortcake. Later, I buy beans and cook them with matooke for lunch **Caregiver 14**<br>*When I wake up in the morning, I prepare for her maize porridge, she cannot take the maize porridge when It is not mixed with millet flour, and I know she cannot take it without sugar so I mix sugar for her, I usually buy buns for her so I serve her and then later go to prepare lunch, when it gets ready I serve her, she does not eat a lot, she is like a little child, she eats very little. But when you prepare rice and fish there she tries to eat somehow and may be sweet potatoes* **caregiver 12**<br>*In the night, I come to check on her, when she goes to bed, I also leave. Then in the morning, I come back to greet her, and give her medicine, I pray with her, if there is no water, I bring it, If the drugs are over, I go and get her more* **caregiver 11**
	Physical, Emotional, and Financial Burden of Caregiving	Stress, exhaustion	"*If I get stressed, it would be because of other reasons, but not the caregiving role*." **caregiver 3**<br>"*There are times she sits and fails to stand so I have to lift her. She leans on a walking stick and I provide more support for her to stand up and go to sleep or urinate. At times even climbing the bed is difficult and I have to help and lift to put her on bed*." **Caregiver 4**<br>"*Because of my ROTOM job, I don't get time to relax.*" **caregiver 10**<br>*"So, I rarely get free time, I work for Monday to Saturday and Sunday I go to church. If I am not in a village meeting, I am in a church meeting, I am a chairperson or a certain cash around that sits every day at 5pm, I don't rest at all. sometimes I need to go to the garden to see how the hired*

			workers are doing, in a week I go like once because of limited time." **caregiver 10**<br>*Maybe carrying him, because I don't have the energy to carry him, I cannot carry him alone, I have to first look for someone to help me in carrying him, to go and bathe him*. **Caregiver 17**<br>*I try to clean him, I turn him but you are really seeing that he is too weak but carrying him is difficult for me* **Caregiver 17**<br>*Yes, whenever we meet, you find that we have a lot to talk about, so I may not even go to work that day. That's why we even hired that maid so that whatever she wants, she can tell her and even if I'm not around she can come and tell my family members. Since I am always home at 4pm for lunch, she can even be able to send for me through that maid –* **Caregiver 7**<br>*There are no responsibilities that bring that much stress.* **Caregiver 8**<br>*There are many challenges, but I have to overlook them. As I mentioned, we are four siblings, but I am the only one responsible for her care. I buy everything she needs, including food; beans, sugar, and posho* **Caregiver 14**<br>*Occasionally, I get some relief if the plantation has matooke. There is a saying, "Orasigara mukyirago niwe arakyihinya," which translates to, "Whoever remains behind bears all the responsibilities." In other words, the responsibility of caring for our mother falls solely on me.* **caregiver 14**<br><br>*Yes, like soiling himself, because every time he does, I have to clean him and change for him clothes*. **caregiver 16**<br>*The truth is I get tired, and because of carrying him most of the time, from the bed to the chair and from the chair back to bed, I got prostate*

			enlargement. Due to over sitting, because I stay in the same room most of the time, I can even spend the whole day without going outside, I developed back pain. When you sit for long, there is need for exercises and I don't have that opportunity to do exercises. So, those are some of the challenges I have found with this role. And maybe there are times when find that I have some sexual desires and there is no way I can leave the old man behind and have him satisfied **caregiver 16**
	Changes in caregiving role	How and what responsibilities have changed over time	*"It hasn't changed, when am to go to the market, I don't cook, I first go to the market and at 10.00 am when I come back I do the cooking."* **caregiver 1**<br>*"Maybe doing some work early before gardening but apart from that we are okay. The important this is to wake up early in the morning and do the work before gardening."* **caregiver 1**<br>"*If I want to go digging, I have to wake up early and prepare her breakfast, then prepare food which I know she will eat and leave the rest for lunch on low heat to keep it warm."* **Caregiver 2**<br>"*Now? I wonder what to even tell you. Now, because of the dementia, he does not tell you anything like, "I want this or I need this", because he is lacking in his normal frame of mind. So, it is up to you to decide for him, otherwise if you sit and wait for him to tell you, you will not manage taking care of him*." **caregiver 3**<br>**"***The added roles include bathing her, washing clothes, cutting hair, nails generally personal hygiene.*" **Caregiver 4**<br>**"***My work changed because I used to make baskets and those would support me financially, I used to do a lot of works but I no longer do them because of this woman."* **Caregiver 5**

			**“***I have to first boil water, then clean her home, for example, like you are here, if you found her in a dirty environment, I am the one to be embarrassed, so I have to clean her home and also clean mine, and it consumes my time, it is not easy to maintain two homes***.” Caregiver 6**<br>*Yes, before she got this condition, I would spend even four days without seeing her, but now I am forced to constantly check on her, even when I am around for like three days, I have to send for her. I cannot spend a week without getting to know how she is doing, yet those days I would even spent a week plus without checking on her* ***– Caregiver 7***<br>*I started a business so that I am close to her.* **Caregiver 8**<br>*carrying him, bathing him, changing for him bed sheets, and cooking for him on time*” **Caregiver 17**<br>*That time she would cook for herself, sometimes she would collect firewood herself but because the disease has progressed, she no longer does all that* **caregiver 14**<br>*Some of the current changes include feeding through the tube. Also, when I came, I could support him to go to the toilet but now he soils himself* **caregiver 16**
	Other challenges	Sleep Disturbance	“*At times she wakes up and keeps moving in the house at night and sometimes forcefully finds me in my bed. Sometimes she wakes up and wants to go out and walk naked and then I forcefully hold her to dress her and put her back to her bed. When I looked at all these, I said I won't manage these conditions, so I decided to keep locking her from her bed room. So when she is going to sleep I put in a bolt on her bedroom door so that in the morning when she is wake I will have woken up already and know how the situation is because if she wakes up and opens for herself then she will move naked.”* **Caregiver 5**

			Yes, I try to rest because I have diabetes, so I must get time to relax or rest like I've come now I'm here to rest – **Caregiver 7**<br>*For people like us, you cannot get over stress, that's why we get diabetes because we don't rest, the mind does not relax, even now like I am here, my mind is not relaxed. The fact is that once you're doing a transport business, your head or mind cannot relax, I can assure you Madam. For instance, you may be there and they tell you the vehicle has got a mechanical problem, or my grandmother tells me, "I don't have sugar", or the children tell you, "Daddy we need fees", so even when you are sited at home, your mind might still be working* – **Caregiver 7**
		Hygiene	"*Even when she tells me she is hungry or that she was not served food, I don't blame the people I have left with her, because I know she might not be telling the truth. If she wakes up from sleep and defecates in the house, I just forgive her and clean up the mess myself. If I asked her to clean it, she could end up making an even bigger mess by littering it everywhere, so I prefer to handle it.*" **caregiver 10**<br>"*In most cases, she bathes herself, but sometimes I help her to scrub her back, where she cannot reach. There are some parts of the body where she needs orders to clean so I have to monitor her when she is bathing. Even changing clothes, she needs orders*." **caregiver 10**<br>"*She can't bathe herself. To bather her is to forcefully do it. She tells you but why do I bathe? And I tell her to look good and not smell for people. She starts arguing that it isn't her problem to be like that, she used to bathe herself. She asks many questions."* **Caregiver 5**

		Diet	"*The eating reduced. She doesn't eat much. In fact, she has only taken two fingers of matooke and it will take some good time before she eats anything again.*" **caregiver 1**<br>***"****Yes, I help him because he has to eat and drink, so you cannot fail to cook for him, because he has no one to help him.****"*** **caregiver 3**<br>"*We are in a tough season, there is need for food and drinks. These are very hard times*." **caregiver 3**<br>"*Yes, when she was old, could not cook for herself, bend to wash her feet. I do everything for her. She put food on the plate and eat or get porridge to drink from the bucket. Where she sits is where she will remain, you have to know that she has not yet eaten so you bring her the food and the porridge. She is like a child and I think that's why she forgets. If someone cannot think of getting food from the sauce pan then that means her thinking capacity isn't as good as before*." **Caregiver 4**<br>"*………... even when you are cooking, and the food delays, she becomes annoyed and then she gives comments like "I used to do this myself, maybe by now food would be ready." In fact, if it was like the daughter in law, she would feel bad and maybe mum would have offended her, but because I am her daughter I don't mind, I take my time until it is ready*." **Caregiver 6**<br>*Now like digging, you cannot dig in time, even when it's like a planting season, you may find when others are ahead of you.* **Caregiver 8**<br>*Feeding is difficult, he feeds through a tube****.*** **caregiver 16**

		Communication	"*She forgets what I tell her but otherwise she is okay. If you say we will sleep without eating, she will not complain. If you give her the food, she will eat it*."<br>"*I have not quarreled with her in all the years we have lived together. I may tell her, "I don't want to cook today and she will say its okay you can leave cooking and we sleep. Then I ask if she isn't going to feel hungry? You realize she is less concerned but you are the one concerned.*" **caregiver 1**<br>"*Like when I warm for her water for bathing but when she reaches, she says it's not hot yet it's very hot. When you reheat it then she says as its very hot."* **Caregiver 2**<br>"*For instance, as I mentioned before with my sister, when she is eating and you call her, she forgets that she was eating and doesn't go back to finish her food, leaving it for the hens. I have to remind her that she was eating. If you give her money, she just puts it aside and forgets about it, making it easy for others to steal. She cannot remember where she placed it. If you send her to buy something, like a shortcake, when she gets to the shop, she forgets what she was supposed to buy. She takes whatever they give her or sometimes even loses the money on her way."* **Caregiver 10**<br>*He has been talking, but now he no longer can be able to talk, he has totally failed.* **Caregiver 17**<br>*When you are conversing with her; she talks the right things but after the conversation, she forgets what she has just said and she can be like; what was I talking about or where did I start, then she will be like, I had really forgotten what we were talking about***. caregiver 13**

			Even when his children come to check on him, if I am not there to explain to him, he can not recognize them **Caregiver 15**<br>*Yes, at times she tells me, for example, when she doesn't have matooke, she tells me, "I don't have matooke", and indeed when I check in her kitchen, I find it finished, so, I bring it for her. But you can also bring it and she forgets to cook, if you do not come to check on her, she can even end up not preparing it.* **caregiver 11**<br>*Yes, when she is stable and you ask her in case you want something she tells you, but sometimes you ask her and she also asks you, "Why are you asking"?* **caregiver 11**
		Medical care	"*I don't face any major challenges in taking care of him, because when he was admitted in "Kisizi" I went with my son. At ROTOM, we were also there with him. So, the biggest challenge would be lack of medical attention, but ROTOM provides the treatment free of charge, so my only role is to take care of meals*." **caregiver 3**<br>"*She is not on any treatment except that I give her foods that can help her boost her brain, we are caring for her, and we ensure that we don't annoy her*" **caregiver 10**
		Making time for pleasant activities	"*Since I am the only one that is around so if I decided that I won't prepare lunch today, would she spend the whole day hungry? I am the one close by so I have to make sure I continue giving her care*." **caregiver 1**<br>I only rest when I go to sleep at night, and I am not used to sleeping during the day **Caregiver 17**<br>One, I have a wife but I don't even get time to go and see her, I find it challenging to leave this old man unattended to. Secondly, I don't have

			the Liberty to take a walk or get a break from taking care of this old man I have to stay close to him all the time the rest are mino**r caregiver 16**<br>I don't move out at night, but at times when I have batteries, I listen to the radio with my mother in the evenings, then at around 9pm we sleep **caregiver 13**
	Coping strategies	How to cope with challenges related to dementia caregiving	"*I was initially scared that I might not manage to look after her but I asked for God's grace to help me.*" **caregiver 1**<br>"*We just had to accept him because we have no solution to the problem.*" **Caregiver 3**<br>"*I usually rest and listen to songs is on Sunday when I go to church. When I go to church I sing listen to the sermon and come. At 8pm I listen to the news."* **Caregiver 4**<br>"*Yes, she washes some parts of her body, and then on Sundays, I warm water and give her a full bath before we go to church*" **caregiver 6**<br>"*One of the things that have helped me manage is love and forgiveness. If I was to use a cane, I would be beating her every day, but I tried to be polite and gentle to her because if I become rude or treat her harshly, I may worsen the situation. I have learnt to accept her as she is because I know that the disease she has no cure."* **Caregiver 10**<br>"*I find it hard to take care of others when mine is home unattended to, and yet the most attention should be given to mine, then others can come in later, I cannot go to counsel others without first counselling mine*." **Caregiver 10**<br>*Even if I am not around, like I am away for two days, I have to ask if she has come around, but whatever happens to her whether good or bad I'm always aware* – ***Caregiver 7***

			You accept the situation, keep quiet and let go **Caregiver 9**<br>*I bathe him once or twice a week, after bathing him, I give him clean clothes, for example, from here I am planning to go and bathe him, if he refuses, I try to convince him that the whites are coming to visit and have to find him clean* **Caregiver 15**<br>We talk, she tells me whatever bothers her, and whenever she needs my help. Sometimes, she tries to check on me, for example, last night, she checked on me at 2am**. Caregiver 14**<br>*……………….. because if it wasn't for it, this place would have failed me. This place is very cold and quiet, it's like a prison, so, I spend most of the time on phone, the good thing they buy for me internet data, so I leave the phone when my eyes are tired and I sleep* **caregiver 16**
		How they stay connected with person with dementia	"*She will tell you. Say if we prepare a certain type of food, she will tell you that that is not the food she wanted. So, you adjust according to her preferences. So, at times I prepare food and she says, "I want greens". So I go in the plantation and get them and prepare for her. If she says she doesn't want to drink from a certain cup. Then you bring the cup she wants but if I don't have the cup, she wants I tell her I don't have that cup so she accepts though it leaves her unhappy.*" **Caregiver 1**<br>*"When I see that she`s annoyed, I move closer to her and tell her we were not talking about her. I assure her that we were in our own affairs then she says, "I thought you were talking about me" and I tell her no and then she returns to her normal self."* **Caregiver 2**<br>"*There is no way we can fail to stay connected. When I am not around my son is always here, I have my sisters in law around, if I am not around, they come in and give him a helping hand. And sometimes when we are*

			not around, this sister of his, can prepare food and bring for him." **caregiver 3**<br>"*When I complete washing the clothes I put them on her bed and when she finds them there she folds them neatly and puts them in her suitcase. At times you find her lifting sauce pans putting them on the rack, plates in a basket, so she does that. So when I ask what she is doing she replies," I am used to organizing my space and things and now they are not organized*.'' **Caregiver 4**<br>"*When we are at good terms, and I talk to her nicely, for example, if I tell her, "Please stay here, I will bring you a shortcake when I am coming back," she becomes happy and keeps expectant, so when I come back she asks me for her shortcake*." **caregiver 10**<br>*Sometimes she gives me advice, saying things like,*<br>*"It will be hard to educate your children if you focus on taking care of me." Although she values education, she is not educated, recently, for instance, we experienced windy rain that damaged all the matooke I had saved for sale. She often reminds me that by concentrating on her care, I may not have enough time to earn the money needed to educate my children*. **Caregiver 6**<br>*You try to convince her and serve her something that she likes, for example, my mother likes cold porridge, so I make for her some and put in her bottle, within a few hours, she finishes it, and then I prepare more for her*. **Caregiver 6**<br>*Yes, at times I spend quality time with her like from 10am up to 2pm or even 4pm –* ***Caregiver 7***<br>*Just spending quality time together, eating, drinking and sharing ideas on different things, if she has come to my home, I accompany her to her*

			home. If at all I am at her place, I say bye to her, leave her with some money like 10,000 and come back – ***Caregiver 7***<br>*Yes, we discuss everything, we don't hide anything from each other –* ***Caregiver 7***<br>*By being patient with her, whatever she forgets you keep reminding her.* **Caregiver 8**<br>When we are talking, sometimes he understands while other times he does not understand, so, when I realize that he has not understood, I just excuse myself and go to do other things **Caregiver 15**<br>Whatever I tell him, he hears and understands me, in fact if I tell him that, "Open the mouth, we are going to brush", he opens the mouth, when I tell him, "We are going to eat", he responds, so he hears what I tell him and understands me. **caregiver 16**<br><br>*We talk, she tells me whatever bothers her, and whenever she needs my help. Sometimes, she tries to check on me, for example, last night, she checked on me at 2am.* **Caregiver 14**
		Relaxation technique from caretaking	"There is no way you can relax and spend a day without eating, that is impossible unless you are fasting yet ulcers patients cannot fast, so I have to cook every day, in other words I cannot relax from cooking. I play my role." **caregiver 3**<br>"*I get to rest at night but sometimes I don't even get to sleep at night because there are times like 2am when she tells me that she isn't feeling well so I go and sit with her. When she tells me that she is feeling much better, that's when I leave her room and sleep*." **Caregiver 2**

			“*I have my phone that has a memory card that has music to relax. So I sit and listen and get comforted and energized. When I feel like giving up after I have done so much work I listen to a song, and it calms me down. Sometimes I also pray for God to guide me on the role I took on and he is the one that provided it so that am able to perform it well. So you find my prayers are answered or sometimes I am energized.”* **Caregiver 4**<br>*When I finish eating at 4pm, I stay at home up to 8:30pm when I go to sleep, wake up at 5am, to what I have to do, at 8am, I go to work, my driving job. But I rest after eating at 4pm* – **Caregiver 7**<br>“*She does not stress me, over these years I have accepted to take her as she is. At first, I was so stressed with this caregiving role, I even tried to use a cane, but later I realized I was wrong, because she was not to blame. I realized she had no control over the things she was doing, so I was the first to change, I learnt to be patient and told the rest of the family members that if she makes a mistake, they should leave her for me to handle, so I just had to accept her as she is*.” **caregiver 10**<br>*I do not relax because I am in charge of everything I would relax if I had someone to help me with some of the tasks but that person is not there it is a matter of being patient with the situation and do everything for her myself* **Caregiver 9**<br>“*There is no way of relaxing because if you have no helper you die on job like one who died from Kyera. “Have you seen it?” if you have no helper you die from the job.*<br>*You see this disease is not only here, it is common this side in the elderly, it has killed three women this side.”* **Caregiver 5**<br>*You read the Bible and get a word that comforts you, and when Sunday comes, you go to church and get some relief.* ***Caregiver 8***

			There is no way you can rest because even at night, sometimes when he is badly off, and you spend the whole night moving up and down, looking for a vehicle to take him to the hospital **Caregiver 17**<br>*You cannot relax when you have a lot to do or when everything needs your attention. You find yourself with a lot of work like; fetching water, picking sauce from the garden* **Caregiver 15**<br>*I do not relax there is a saying, "Orume rwakura ruronka abaana" which translates to "When a rabbit grows old, it relies on its young ones.", in other words, as parents age, they become dependent on their children for support and care. So, I take care of her as my wife, because if soap, jelly, etc get done, I have to provide more, I have to try by all means to get money, the expenditure is more than the income* **Caregiver 14**<br>*It's just praying to God* **caregiver 16**<br>*For the radio I only listen to the news in the morning and in the evening* **Caregiver 12**<br>*I cannot relax because it is my responsibility to take care of her, I was given the responsibility of taking care of her by ROTOM* **caregiver 11**
		Creating time for pleasant activities	"*On Sunday when I haven't gone to the gardens. That's when I cook, wash clothes and go to church*." **caregiver 1**<br>**"***I usually rest and listen to songs is on Sunday when I go to church. When I go to church I sing listen to the sermon and come. At 8pm I listen to the news."* **Caregiver 4**<br>*They also help in doing the routine responsibilities of taking care of her like bathing her, washing for her and cooking for her.* **Caregiver 8**<br>*Usually, when I have finished all my work, I sleep for like 30 minutes before waking up to do other things where I can get some earning. I did*

			*not go to school, so I do not have any formal employment, I have to rely on my hands to earn a living***. caregiver 14**<br>*It is not possible to rest during the day because I have a lot to do and time for resting is not there. Because you have wake up early and do some few tasks for her, then you have to go to the garden to dig. You do not even get enough time to complete this task, so by the time it reaches 4.00 pm I am releasing the goats to graze in the fields* **caregiver 13**<br>*When I come back from the garden, I bathe, I have a Bible on my phone, so I start reading the Bible, in the evening, I move to Muhanga town, where I pass time until evening when I return home***. caregiver 11**
		Involving others in caregiving roles	"*When we don't have food to eat. I call my elder siblings and they help me. Then at times I wake up and we don't have anything to eat like you know life in the village but I call and they are able to support us*." **caregiver 1**<br>"*Ms. …….. (name with held) tries a lot to provide assistance. When I tell her grandmother isn't able or is sick in anyway. She counsels me gives me money to take her to ROTOM. If I ask, "as grandmother is forgetting more often", then she buys pumpkins to cook for her to improve the situation."* **Caregiver 4**<br>"*My mother comes say a week. She bathes and applies Vaseline, clothes her later goes home because she has a family. Then also the one who stays in Kampala when I tell her we don't have soap or Vaseline, she knows the challenges here so she sends the money for both and at times sends the clothes for grandmother."* **Caregiver 4**

			"*Whenever I want to go somewhere, I have a neighbor and she is also her in law so if I decide to go I prepare food and porridge and tell her to give her lunch when am going far and she helps me.*" **Caregiver 4**<br>*"I am the one who takes care of her, if the visitors come they maybe give her just little money like for short cakes, I usually have soap for washing, for food, we get from the farm."* **caregiver 10**<br>"***Laughs*** *whom do I tell? It's my burden to die with. You know if you tell someone about your bad condition, it's just a tale to them, they don't have you at heart or even comprehend what you are talking about. Yes, but because of dementia, I had children, but because of dementia children ran away and also her grandchildren. I remember Mr. I what you were teaching us about and it has come to pass ooh God (Laments). God have mercy, my wife doesn't want to see any child, I always ask her if she doesn't want to see children, then why she gave birth to them. How does God give a gift and you refuse but we have accepted it. She has refused children who would have been of help to me. God alone will be my strength."* **Caregiver 5**<br>*Yes, I do for example when I prepare our supper I put some food in a dish and send one of my children to take for her by doing that I am relaxing*. **Caregiver 6**<br>*Explaining to them to get used to her condition.* ***Caregiver 8***<br>*No one helps in doing that, everything is entirely my responsibility. I do everything for her* ***Caregiver 9***<br>*When they are around, they help in giving him tea, checking on him, in fact now that we are here, some are at home cooking, so that they keep bringing for us tea and food including; breakfast, lunch, evening tea, and supper* **Caregiver 17**

			*She prepares for him when I am not around. She told me that since I'm always away looking for money, she can always help with lunch so that I prepare suppe*r **Caregiver 15**<br>*There is a son of his that we stay with, I usually tell him. He is actually the one who usually takes him to "Muhanga" (ROTOM)* **Caregiver 15**<br>*Yes, I ensure that even when I'm not around, I send my wife to go and check on her* – **Caregiver 7**<br>*Apart from ROTOM and I, she does not get help anywhere else, except one daughter in law who stays in the neighbourhood* – **Caregiver 7**<br>*I have always told them, but they don't seem to care. Even the chairperson of this cell knows that I am the one taking care of this old lady. You could ask anyone in the village, and they would tell you that everything concerning her is my responsibility. If I was not here, she would not be either. So, I will continue to do my best until I can't anymore, then maybe ROTOM would come in to help. As long as she has treatment, my main worry is providing her food.I don't have reliable relatives anymore; the trustworthy ones are from the past. These days, even a relative can turn out to be an enemy.* **caregiver 14.**<br>*I call them where necessary, whatever is required, I call them and they act, in case of need for clarification, I call the doctor here*. **caregiver 16**<br>*There is one of the grandchildren, when they come for holidays, he spends with him like four days, so, during that time, I go home* **caregiver 16**<br>*There are nieces and nephews who come around, and at times they help me to fetch water or collect firewood; in fact the one you sent to call me, is one of them, they are even twins and they are for my young sister* **caregiver13**

			When I want to go far, my older daughter sometimes helps me to prepare porridge for the grandmother and serve her. There are times when I call her daughter to come and attend to her **caregiver 12**<br>My wife can help in preparing food but the biggest part of care is provided by me. **caregiver11**<br><br>There is my young brother, he has been helping here and there, but you know issues of siblings, sometimes, they tend to refuse to do something so that the other one can do it, but the ROTOM responsibility was entirely given to me. They emphasized that it must be done by one person, because with two people, the patient may suffer, at times, she can go without drugs or even spend a day without food. So, the responsibility of taking care of her is solely mine **caregiver 11**
	Interacting with someone with dementia	Communication strategies	"*Yes, we try to talk to him gently that, "Allow us to assist you with this*." **Caregiver 3**<br>"*She laments and asks if she will be normal again like other people and stop using the walking stick. She is getting old but thinks like a young person, she thinks she will be able to dig like other people. She doesn't know she is growing older but younger or wishes it was so. So that she is able to go for church but now she isn't strong enough to go to church. When she wants church then we tell the priest to come and visit her, pray for her, gets holy communion which makes her happy."* **Caregiver 4**<br>*You try to convince her and serve her something that she likes, for example, my mother likes cold porridge, so I make for her some and put in her bottle, within a few hours, she finishes it, and then I prepare more for her.* **Caregiver 6**

			Yes, before she got this condition, I would spend even four days without seeing her, but now I am forced to constantly check on her, even when I am around for like three days, I have to send for her. I cannot spend a week without getting to know how she is doing, yet those days I would even spent a week plus without checking on her – ***Caregiver 7***<br>*Even if I am not around, like I am away for two days, I have to ask if she has come around, but whatever happens to her whether good or bad I'm always aware –* ***Caregiver 7***<br>*As long as we discuss things that make each other happy, then we remain happy, but when we are discussing things that offend the other person, then you have to become angry and offended but you have nothing to do about it****. caregiver 13***<br>*I keep doing the usual responsibilities. We talk normally about so many things, we have no problem* ***Caregiver 9***<br>*Whatever you talk about, by the following day she will have forgotten everything* ***Caregiver 9***<br>*We talk to her and communicate normally, only that by the following day she will have forgotten whatever you discussed.* **Caregiver 8**<br>Whenever he wanted to do something, he would call the whole family and tell us **Caregiver 17**<br>*Because she loves me so much, sometimes she comes to check on me very early in the morning, and there are times when I come to check on her* **caregiver 14**<br>*Whatever I tell him, he hears and understands me, in fact if I tell him that, "Open the mouth, we are going to brush", he opens the mouth, when I tell him, "We are going to eat", he responds, so he hears what I tell him and understands me****.*** **caregiver 16**

			When she wants something, she tells me, then it's me to tell the daughter **caregiver 12**
		Activities the patient wants to continue independently	"*She would want to go in the plantation to weed and she will try but after like a few minutes she will become weak and sit but you realize she wanted to work but felt weak*." "*She would like to do everything herself. You find her with some fire wood that she is from collecting firewood and gets a small jerry can and fetches water so you realize she would want to do the chores by herself*." **caregiver 1**<br>"*She liked digging and washing her clothes. At times I would wash for her and she says the clothes are not clean and would want to repeat the laundry by herself but she can't.*<br>*She liked going to the market and to sell cow ghee. You can see that she really wants to do it but her energy fails her*" **caregiver 2**<br>"*Sometimes he does not like it when we offer to help in washing, especially the clothes he puts on for Sunday service, because you may not wash his clothes the way he prefers, so he washes for himself, and puts his shirt on a hanger, and then keeps his shirt away*." **caregiver 3**<br>"*Even digging, if it was possible, he would have loved to do it, but he cannot manage because his shoulder got a fracture and he can't use it. However, he usually talks about gardening and wishes he could be able to partake*." **caregiver 3**<br>"*She would live to dig, she at times requests for a small hoe and does some little gardening around the house with her walking stick. She may not succeed till the end but she will do something. Gardening in the*

			compound, she will use her panga to weed the compound. That shows she would have loved to do work herself but she isn't strong enough." **Caregiver 4**<br>"*Cooking and organizing the house to make it neat but it's not possible*." **Caregiver 4**<br>*Most of these elderly women do not like their daughters in law to bathe them. They never want them to see them naked, you know that is a private area. In fact, even when I am bathing her, she does not allow me to wash her private part, she washes it herself, and then call me to dress her up*. **Caregiver 6**<br>*She would have preferred to cook for herself, even digging, she would love to do it, because even when she hires someone to dig for her, she makes sure that she goes to check on that person. But her health is not well that's why she no longer cooks or even digging – **Caregiver 7***<br>*Digging, washing for herself .* **Caregiver 8**<br>*Taking care of herself and do everything by herself* **Caregiver 9**<br>*The time he had the strength, he liked digging, he would dig, harvest sell and get money, there is no season that would bypass him. But when he fell sick, he stopped**.*** **Caregiver 15**<br>*when bathing, she feels like you shouldn't touch her* **caregiver 13**<br>*Yes, if she had the strength she would be bathing herself* **caregiver 12**
		End of life discussion	"*She usually says if we don't stay close to her, that one time we shall find her dead.*" **caregiver 1**<br>"*Even if the disease progresses, and his health deteriorates, we shall continue being patient with him until his death, because he has nowhere else to go and no one else to take care of him.*" **caregiver 3**

			"*He usually says that, "I wish I could just fall asleep and God helps me and I never wake up.*" caregiver 3<br>"*She says her house should stay and I keep looking after her property. She doesn't want to be taken away and suffer from somewhere else, she wants to remain in her home till death.*" **Caregiver 4**<br>"*You find her saying, "I am going to die and you marry and you have your wives, do I still look important to you?" And I ask her if she has ever found me sleeping with a woman? Laughs, and I old age is a disease if I were still young I would show her. It leaves me wondering how God created us, even at her age she feels she needs to be with a man*." **Caregiver 5**<br>*She said that when she dies they should make sure that they cement her grave. She also said that they should burry her next to the husband's grave.* **Caregiver 6**<br>He used to pray like, "God, I wish you can take me now".**Caregiver 16**<br>*Of course, their future is hard, because she may come to point where she may not be able do anything for herself or on her own. She may also get tired because even when it comes to the person taking care of her, she/he may not give her all the attention she desires. Such people need or require close attention, something that is impossible –* ***Caregiver 7***<br>*She usually asks me one question, "What would I do if you were no more"? She asks me that question every day, that, "What would I do without you"? Even when I got diabetes, she told me that, "Although you are sick, I pray that you remain alive". Even when I get something bothering me, I cannot tell her, because when I do, it bothers her to the extent of crying. At times when I am sick, I don't tell her, I have to hide it from her. In fact, like now, I am sure she knows that I am around, because the other day she got me some herbs for steaming. I have been*

			having flu, she told me to steam instead of buying medicine from the pharmacy, that I would be well after steaming – ***Caregiver 7***<br>*She has a son who spends long without coming to check on her, that is why he is just constructing yet he is about 50 years of age. I am even the one who forced him to buy that piece of land where he is constructing. He spends most of his time in Kampala, so realistically you also see that it is not possible to get immediate help from someone in Kampala –* ***Caregiver 7***<br>*Yes, she gets bothered so much. In fact, now that he is constructing, she is a bit relieved, but before he constructed, you would find her lamenting and wondering why he cannot even have a house there –* ***Caregiver 7***<br><br>*She doesn't mind about her end of life, she just tells you she's waiting the final hour, that's what she usually tells us, that, "I have finished everything I had to do in the world, I'm just waiting for the final hour".* **Caregiver 8**<br>*She usually says that, "I am old, in fact, I don't see the incense of going to the hospital* **Caregiver 9**<br>Yes, he actually wrote his will, and distributed all his property amongst his children, every child knows what he or she owns. He did this while he was still talking. **Caregiver 17**<br>Yes, there are times when he says that "If I died now, where would I leave my relatives or my children, or how would they survive without me"? **Caregiver 15**<br>So, I usually tell him that whoever will be alive by that time, will see how to handle them **Caregiver 15**

	Support	Type of support, From who	"*My sister at times comes and also washes her clothes. My brother took on the responsibility of always buying the soap.*" **caregiver 1**<br>"*At times when I feel too tired to dig they send a laborer to help me dig.*" **caregiver 1**<br>"*There was a helper before but her conduct wasn't good so I decided to take on the role of care giving myself*." caregiver 1<br>"*May be get us food supplies but the rest of the work we can do.*" **caregiver 1**<br>"*She likes food and drinks a lot. So at times she would prefer meat but I have no money. So one has to garden and when I have harvested I can be able to sell and buy her eating preferences.*" **caregiver 1**<br>"*No, if one knows they have a mother out there and do not bring for her soap, am I the one going to ask it from them. That means they may also not be financially well at the moment so I try to do my best and when she says she wants something, I may not provide it that day but I try when I get money and provide.*" **caregiver 1**<br>"*There are people that help me like when I am feeling weak; there is someone that comes and washes for me and also cooks.*<br>*There are some that give me sauce especially since we were hit by drought and the yields were low so they bring me sauce and eggplants, that's how they assist me."* **Caregiver 2**<br>"*The aid we would have needed is quite a lot but most importantly, the needed assistance is in provision of food and drinks for him*." **caregiver 3**<br>"A helper would be welcome if possible" **Caregiver 4**<br>"*I would wish to have someone who can help me stay here with her, for example, if you left her around, even if a thief came to steal, he would*

			take everything when she is watching. In fact, when I leave her here alone, I close her inside the house, it would be like I have not left anyone at home." **caregiver 10**<br>*I am the one in charge of taking care of her, even her sons, none of them can take care of her, except this one son of hers, who brings some maize flour for porridge once in a while.* **Caregiver 6**<br><br>*Maybe washing and cooking* **Caregiver 15**<br>*Bathing him, washing Anyone who could help* **caregiver 16**<br>*If it was possible, I would prefer to get her someone to stay in this house with her, because my house is the other side. My father is a drunkard so even when he returns, he does not bother to ask her if she is well, he just enters his bed and sleeps, the following day, he leaves, in fact, you are lucky to have found him around at this time of the day. So, if there was money, I would have hired that person to stay close to her, so that in case she gets a problem at night, that person can inform us***.** **caregiver 11**
**Training**	General information	Views about training	"*I would happy and when you teach me and encourage me. I would get energized*." **caregiver 1**<br>"*One needs to be trained, to learn and understand*." **Caregiver 2**<br>"*If a training was brought then I would attend so that I can learn something I may not be knowing on how to take care of grandmother. I would need the training because there are some things I may not know so it help and improve on the care I provide*." **Caregiver 4**<br>*…because it is necessary to learn how to deal with these elderly people, for example, how do you take care of them or if something is bothering them how do you convince them to remain calm* – **Caregiver 7**

			I usually ask her softly like, "What would you have preferred?" and she tells me, for example, "I would have preferred goat meat instead of beef", then I buy it for her and she becomes happy – **Caregiver 7**<br>*You try to trick her, for example, if it is washing you bring a basin and maybe put a few clothes like two, she tries washing and that makes her happy, because she feels she has done it herself and done it better. Although she no longer has the energy, she sweeps the house herself, because she is a clean person* – **Caregiver 7**<br>*She requires a constant supply of some items like; soap, sugar, even money, if she has like 20,000/= in her pocket, it may make her happy. When I come around to check on, I usually give her some money like 30,000/= as she is going back and it makes her happy.*<br>*Recently, she was telling me, "ROTOM brought for us some special piece of cloth, and it can only be acquired after paying 15,000/= yet I don't have money" she cried and was like "Am I the only one going to be left out"? so when I gave her 20,000/=, she was very happy. So, if she has some money with her, even if it's just 30,000, it may make a difference. I am sure as a person, there are things she desires to sort but feels as if she is stressing me or as though she is a burden to me, meaning she may decide to keep quiet* – **Caregiver 7**<br>*Learning never end, I am willing to attend only that if it happened like tomorrow, it would be hard for me to attend, because as you can see, I can't leave this old man alone.* **Caregiver 16**<br>*I would be willing to attend, except if it starts early and ends late. There I would fail to attend, because if it ends late, I would find that goat still in the bush and it is actually one of the stressors for her. If it starts very early*

			I would fail to prepare anything for her, so I would end up missing some parts of the training **caregiver 13**<br>*I would love to attend the training, Bakiga have a saying that "Omukiga aratiganda mukanwa kamugyenzi weye", literally meaning that, "We learn from other people" so I would be glad to learn from others so as to get the skills to take good care of my mother. So that I learn things like; if she is sick you should do this, you should turn her like this, you are supposed to handle her this way, something of the sort* **caregiver 11**
		willingness to attend	"*I would come. I would come and learn what I didn't know to improve on the care am giving her."* **caregiver 1**<br>"*Yes, I would like to attend*" **caregiver 2**<br>"*If it comes, we would accept it and attend*." **caregiver 3**<br>*For the training, even if I am not around, I would send someone to represent me…* **- Caregiver 7**<br>"*If the training becomes available, I would attend to gain skills that would help me care for my patients and also teach others I interact with in my daily work.*" **caregiver 10**<br>"*I can join it."* **Caregiver 5**<br>"*Yes, I would attend*" **caregiver 6**<br>*Honestly, I would attend.* **Caregiver 8**<br>Yes, I would attend **Caregiver 17**<br>*Yes, if the training was there, I would be glad to be part of it to learn how to take care of my husband***. Caregiver 15**<br>*If they call me I would attend and learn like others, because you cannot fail to learn something from such a training* **caregiver 14**<br>*Yes, I would love to attend with others* **caregiver 13**

			I would attend **caregiver 12**
		Content desired	"*How to take care of them. So one reflects back at home and decides best on what to do. If the old woman has her habits then you can talk to her on how to behave according to the training so that they know what to do*." **caregiver1**<br>"*There is a lot to learn; like taking proper care of your patient, his personal hygiene, his feeding and nutrition but you find that you do not have what it takes to take care of your patient, but you find that with the little resources you have, you are trying.*<br>*So, there are many kinds of training, including the one for providing skills, animal rearing for farming purposes, so that your farm produce can cater for your patient, etc. Things like that.*" **caregiver 3**<br>"*I would like to learn about the causes of mental disorientation, and guidelines for taking care of a person with mental disorientation.*" **caregiver 3**<br>"*I cannot say that I want to learn on this specifically, because currently, I am using basic knowledge to take care of her, I am not a skilled caregiver, so I would wish to attend the training, and whatever you teach, I am willing to learn, so that I gain skills of taking care of her*." **caregiver 10**<br>"*I need training in how to take care of such people in our homes and in any other way. So that I know how she behaves, how to care for her and the like.*" **Caregiver 4**<br>I would wish to learn about everything concerning taking care of my husband and may be the things I'm supposed to do at home, general care. **Caregiver 15**

	Barriers	Possible barriers to attendance	"*If it is near, I would attend*" **caregiver 3**<br>*Maybe if I am sick, it is the only thing that can stop me from attending.* ***Caregiver 8***<br>*I move a lot, because most of the work I do is not here in Muhanga, I go wherever they call me, because I transport or supply sand and stones for construction, so I may be here and they call me to work in "Rukungiri", for example, tomorrow I will be travelling to "Isingiro", I have just come back from "Kisoro", so the training may come at a time when I am not around –* **Caregiver 7**<br>*Because I have a lot to do, I don't have time* **Caregiver 9**<br>*Yes, I would be willing, but the truth is I cannot manage attending the training because I have a lot to do like digging, cooking food because I am always up and down looking for money taking care of this and that in fact, I am waiting for this interview to end so that I go to the garden.* **Caregiver 9**<br>*Maybe if I failed to get someone like a house help or someone to help with taking care of him, but if I got someone to stay with him, I can try and get one to leave behind, there are those who are temporarily. If I fail to get one, I can call one of my sisters to be with him as I go for that training* **Caregiver 17**<br>*I would wish to learn on medication, because the rest I know, for example, bathing him, feeding him, making him talk and massaging him. So, I need knowledge on medications, so that I know if he is in this condition, what kind of medicine am I supposed to give him, how to dress the wound, once I learn that, would feel complete***. caregiver 16**

			Nothing. It is me to benefit from the training so why would I refuse to go there **caregiver 11**
	Resources	Information needed to improve caregiving role	"*You can teach about nutrition and hygiene. For hygiene, for example I may come to your home and admire everything there, but cannot afford to put my home at the same standard as yours. Likewise, on nutrition I may not be able to afford the ideal diet, but when actually I cannot afford*." **caregiver 3**<br>"*Cooking, washing, bathing her*" **Caregiver 5**<br>"*They would find me here because I would not want to leave her here. They teach how to behave towards her, looking after her, hygiene and care in totality."* **Caregiver 5**<br>"*I would want to learn about general care for these elderly people*." **Caregiver 6**<br>*Most of those who take care of the elderly, do things their way or in an ordinary manner, but if you have attended a training you may do things a different way maybe a better way – **Caregiver 7***<br>*Forgiving and love. When you learn to love her, you also learn to feed her and she gets good health. Then cleaning for her so that she stays in a clean place, things of the sort.* **Caregiver 8**<br>*Really, can those ones be taught, okay you know better what to teach which can help us to care for the old people* **Caregiver 9**<br>If we can get people to teach us and they counsel us, we can get hope **Caregiver 17**<br>*I would wish to learn about everything concerning taking care of my husband and may be the things I'm supposed to do at home, general care*. **Caregiver 15**

			I am ready to learn whatever they teach. **caregiver 14**<br>*Whatever they teach, I am ok with them***. caregiver 13**<br>*My mind is open to learning whatever they teach me about during the training is what I will go with.* **caregiver 12**<br>*I have been taking care of her my way, so I do not know if it is right or wrong, but from the training, I can learn how to do it the right way. Like the way I told you that at times I leave her to wake up at whatever time she feels like, for example, 10am, but maybe from the training, you may tell me that it is wrong, that maybe I am supposed to wake her up, since it may affect her health, and make her weaker, or you can tell me that I am supposed to let her sleep until she feels like waking up, something of that sort. So, we are willing to learn from you, whatever you teach us is what we shall go with, just like the way you get ill and run to the doctor for a solution, it is same way we are waiting to learn from you to improve our skills***. caregiver 11**
		Resources/ materials needed to improve caregiving	"[Regarding provision of reading material] *We don't know how to read*." **caregiver 1**<br>"*I do not know how to read so the training materials would do not help*." **Caregiver 6**<br>*When someone is already old, they require a lot of care and the person taking care of her, must know what to do or how to handle, because there is food fit for the elderly, like nutrition, if anything changes, they should rush to the hospital. So, an old person usually requires to be well all the time, because you may be with her now, and within no minute, her*

			condition changes. That is why we got her someone to take care of her, or stay with her, so that if anything changes, or if she is not feeling well, she has someone to tell and this person can give us a phone call or she is rushed to the hospital – ***Caregiver 7***<br>*Teaching me how to handle, because you cannot know everything, but when you show someone how to do things, the caregiving skills may improve.* **Caregiver 8**<br>*It would be ok to leave behind the training materials, but there are people who do not know how to read, but if there is like a workshop, and people attend in person even after that training, you can still give them those training materials and they go with them* **caregiver11**
	Delivery format	Preferred format of delivery	"*They can call us as a group and share experiences and we give knowledge on how to take care of our elderly*." **caregiver 1**<br>"*I would love to learn in a group of other people*." **Caregiver 2**<br>"*It depends; you can choose to call the participants and train them as a group, then we meet then and train them in a workshop.*" **caregiver 3**<br>"*I would prefer an in-person training because if they give me the training materials I would not have an opportunity to ask questions for clarity*" **caregiver 10**<br>"*If called by an organization we sit as a group because there are other people in similar situations. There are others who may have major challenges compared to me so we all learn how to give care to our people.*" **Caregiver 4**<br>*I would want to attend in a group.* **Caregiver 8**<br>*It should be like a group and they call them or teach them in a class, so that everyone understands what has been taught, how things are done, because when it is a group, everyone is attentive and listening to what is*

			being taught. Someone may be given a training material and they don't even read it – ***Caregiver 7***<br>*The best would be in person training, so that we meet other people and we share love and ideas* **Caregiver 17**<br>*I would prefer to attend in person so that I see that trainer and they teach me because for the training materials I don't know how to read and write* **Caregiver 17**<br>*I prefer an in-person training* **caregiver14**<br>*If they bring me the training materials and I read theory without seeing the practical part of it, it would still be hard, I would prefer to attend in person, and learn from the trainer, for example, if he says, "You go and he puts a sign showing that you go", I would learn better, but giving me a lot of notes to read would be hard.* **caregiver 16**<br>*Yes, I would love to attend with others* **caregiver 13**<br>*I would prefer attending with others because when you attend with others you learn from them Instead of the training materials that you read alone* **caregiver 12**

Key informants

**Main domain**	**Thematic area**	**Sub Theme**	**Quotes**
Knowledge and skills	Perceived knowledge and skills of informal caregiver	Common misconceptions	"*Yes, the first of misconception is that they normally take dementia as it is bewitching, they take it as maybe someone has bewitched they doesn't know that it is also a disease like the rest of the diseases then another misconception they normally have like that is the most misconception they normally have traditional witches and what*" **Key Informant 1**<br>**"***Some few don't believe like it needs medical attention others believe it does because maybe the drugs help them then others I think they get tired because it's something that it will not go away, then others feels like its normal that's how jajers are,"* **Key Informant 2**<br>**Bewitching**<br>**"***Not so much but you know like African tradition in case of anything unusual or what maybe something maybe someone is doing something spiritually to them but in this region also I faced that so much in Mukono the time I worked in Mukono I think those bagandas over believe in those things but this side not so much though some also believe maybe there is something not right maybe just a mental issue but not so much like the other region***" Key Informant 2**<br>"*Ooh yes there was one case of course people think of someone has been bewitched there are people who get early onset of dementia there is a lady who had I think she was like 65 and we didn't expect her you know to develop hallucinations but the lady had been involve in road traffic accident so later I think of course dementia combines a lot of illnesses that have to do with cognition impairment all those things so some thought have been bewitched by neighbour so those are the misconception we have received but we try*

			telling them that is not witchcraft, that this thing is real it comes as someone is aging although is not a disease for elderly our immunity is not compulsory when you become elderly you have to have dementia it happens in some elders." **Key Informant 3**
		Knowledge lacked	*"Maybe first of all since they don't know that they have little knowledge of what dementia is ...."* **Key Informant 1**<br>"*The point of information like the only information they lack is sometimes they lack enough knowledge because it is through their fellowship it is only through when their caregivers have trainings we normally talk about dementia and sometimes since we have a lot of condition to talk about you may find that dementia has been maybe talked once in a year the knowledge to be updated on the condition*" **key informant 1**<br>**Lack of knowledge on what dementia is**<br>*"I think for me majorly since they are not learnt most of them don't understand that it's a medical illnes*s" **Key Informant 2**<br>**Causes of dementia, Signs and Symptoms of dementia, Drug adherence, "** *Like I said we need to continue health educating them on how to pick a few signs and also to tell them that sometimes its normal with age and when it comes with illness there is still medication. Yeah, we try to health educate of course but different topics but they also have their myths, its hard to get it from their heads for them they know, I think that's it*" **Key Informant 2**<br>**"***First of all they don't have the insight you know that this person really has cognitive problem you find them arguing with dementia patient we tell them remember this person has issues with the brain cells so even if the person abuses you have the insight to know that the person has dementia the person definitely annoy you so those are the few things we have been going through*

			we normally teach them about dementia so that they can know that the people they are taking care of need assistance for most of their times" **Key Informant 3**
		Practical skills needed	**Management of bedridden patients and Communication**<br>"*The practical skills they need, first of all we normally tell them that one they need to know how to take care of the because what we have been teaching them is how to take care of the bed ridden patient and what we normally tell them at the as principals of taking care of bed ridden patient to make sure that you are able to turn the patient every 2 hours to avoid bedsores it is one to make sure that since the patient is not going to probably even forget that she is supposed to feed you have to force the patient to feed otherwise the person will die of hypoglycemia or hunger so the critical skills really is to know in terms of communication between each other between care taker and the dementia victims know how to communicate to them, if someone is aggressive don't respond in aggressive manner because when you respond in aggressive manner the other person might even decide not to take medication so you have to respond in a calm and cool way so we teach them about communication skills we teach them about medical skills of how you can really manage*" **Key Informant 3**<br>**Communication and Self care**<br>*"I think they need, most of them lack communication and actually self-care that is what they lack, so that's where they need a lot of training."* **Key Informant 3**<br>**The right time to administer medicines**<br>*"And also the drugs maybe if there is a way or to that can help on knowing what drug to give when and how like to make it simple for them if like chart how they write evening oba night because most of the patients who have*

			dementia are patient who have other illnesses it's rare that it's just dementia alone so we even don't know so for us like And also the drugs maybe if there is a way or to that can help on knowing what drug to give when and how like to make it simple for them if like chart how they write evening oba night because most of the patients who have dementia are patient who have other illnesses it's rare that it's just dementia alone so we even don't know so for us like " **Key Informant 2**<br><br>*"Task, bathing them and then also easing them .those ones they struggle" And also doing number 2. cleaning themselves* **Key Informant 2**
Challenges and support needed by the informal caregivers	Informal caregiver challenges	Common challenges faced by informal caregivers	"*Maybe first of all since they don't know that they have little knowledge of what dementia is because we all know that dementia is a chronic illness that wont be cured in one day or two days sometimes they can get tired of taking care of them because they complain that when they are taking care of those seniors other people they are doing other jobs like looking for what to eat so sometimes they may face the challenge of food since most of the times they are taking care of those seniors*" **Key informant 1**<br>Regarding finding time for their own activities "*Yes, it is a challenge to them because as you know you have to their day living have to go on to look for what they eat to get all their basic needs they have students which are studying they have to also provide their basic needs for their family so if they are most of their time to take they care of those patients it would be hard for them to stay every time on the patient so sometime they leave the patient to go outside to get what to eat*" **Key Informant 1**<br>"*Maybe those ones that we have already seen like the patients not sleeping at night and sometimes you know it is hard even if you are staying with*

			someone who has forgetfulness who sometimes become talkative you can have that stress” **Key Informant 1**<br>**“***I think for me majorly since they are not learnt most of them don’t understand that it’s a medical illness, most of them feel attached to traditional that maybe there is something wrong with this person, some few don’t believe like it needs medical attention others believe it does because maybe the drugs help them then others I think they get tired because it’s something that it will not go away, then others feels like its normal that’s how jajers are, so you find they are not helping or they even neglect I think of getting tired of taking care of but the thing is not going you find that they really neglecting them in helping them in day to day activities like any other chronic illness***”** **Key Informant 2**<br><br>**Overburdened, stress, sleep disturbances**<br>“*Of course, they complain sometimes, we have people like admitted like two here you find they are like they want to do in turns but they are also tired but they are tired no one wants to stay here*” Key Informant 2<br><br>**Aggression by the patients, Heavy Carrying of patients, Poverty, hygiene**<br>**“***Well, from what they tell us that patients, sometimes become a bit aggressive and actually refuse medication so now eventually when they refused medication their condition worsens so they face a challenge of patients being aggressive, they faced a challenge of because they easily forget sometimes they give medication they think they have actually taken it yet they have thrown it away the other challenge they face is some of them who have actually some of them who have undergone late phases of*

			dementia some of them are immobile, some of them are actually bed ridden so eventually they use a lot of effort to work on them these care takers are poor they barely can afford to make ends meet so you find you have to turn the patient two hourly, you have to feed the patient, the patient are no longer able to feed yet previously use to feed themselves you find you have to take the patient to the toilet reminding the patient not to soil themselves from there, so they faced a lot of challenges including hygiene, including drug adherence and so many other challenges **" Key Informant 3**<br><br>**Sleep Disturbances due to overworking secondary to few caregivers**<br>***"T****hey actually do and most of the care takers if you have notice most of them are female I don't know if you have notice that so most of the time you are talking if handle sleep, they don't actually sleep because they have to keep on turning the patient every two hours those who undergone late phase of dementia they have to keep on changing the bedsheet so literally they overwork themselves to be honest and then how they are handling sleep is really we just leave to the lord there is no other option most of the time you find each patient has one care taker its not like they have so many care takers its always one care taker one patient so we have not help them in terms of that they just try their level best so it has really been a challenge for us they overwork themselves some of them have given up and left the patient there so those are the challenges we are facing at the facility***" Key Informant 3**<br>**"***.... but majorly they are struggling with areas of maybe income security because they spent most of their time with the dementia patients so there is literally no time for them to go and make money and then apart from the fact that she doesn't have time to go to the garden to have food, food security for*

			dementia patients food security even for family members because they tend to spent most of the time with the dementia patient since they are very needy and need assistance all the time **” Key Informant 3**<br>**Caregiver burden**<br>**“** *I mentioned most of these elderly people have retired they don’t have money they cannot afford to pay the bill, they cannot afford go houses, they literally cannot afford anything good so it is the care takers who almost takes care of them 100% so they are really going through a lot and by putting it out there really help us* **” Key Informant 3**<br><br>**Lack of necessary equipment for hygiene**<br>*“They struggle they don’t have the necessary equipment like now hygiene it reaches a point where someone need pampers but can’t afford some really can’t afford so it all become nasty just disorganizes the whole thing”* **Key Informant 2**
		Common caregiver questions and concerns	**Food, Forgetting**<br>“*The most of the concerns, they are always asking for food, most of the time they are taking care of the patient and sometimes they are always complaining of those seniors they forget easily so it is hard it is our duties as health workers to explain that somethings they won’t go like that it’s a matter of giving it time*” **Key informant 1**<br><br>**Caregiver burden**<br>**“** *Of course, they aske like will the patient stay like this forever like is there hope of.. I always tell them with medication, atleast the patient can somehow stabilise , I think thts what they usually ask…………..* **” Key Informant 2**

			"*What they normally tell us the question they normally ask is dementia curable that is something they have been asking us, we tell them is not curable we only give medication to reduce the symptoms not to worsen it and then the other question they normally tell us actually not questions but they usually come saying that of course the challenge they meet is there is a bit of burden they are overburden and some of them have lost home, we have what we call home care centers which facility build for them we have one which the facility has built so that we can bring them and take care of them ourselves at the facility we put nurses, we put social workers, we put people there so they have been asking can we bring our people here so that we can remain here apart from is dementia curable and some of them have been promising to escape from their care takers its quite so sad but care takers escaping from their patients you know when some has become incapacitated not able to move people tend to neglect them so you find someone had a family of 12 children but now currently the one taking care of the person is the in-law, a wife to the son. So those are the question they normally bring"* **Key Informant 3**<br>**Desiring an explanation of the illness**<br>"*Of course, they ask the doctor what could be the reason why this person is like this I explain to them some are illnesses, some is just age and they try to understand* " **Key Informant 2**
		Areas where caregivers struggle	*"It is that point of forgetfulness you know when you are staying with someone who always forget and most of the time interrupt things you may find they are always complaining about how those people they are living with"* **Key informant 1**

			**Early detection of dementia signs**<br>"*Maybe as they struggle at initiation they are not aware how to see the signs like to know it early I think they struggle they are not informed or what so most of the people you get have been with it for some time* " **Key Informant 2**<br><br>**Nutrition, Hygiene**<br>*"... so you find they are really not doing well so things to do with nutrition really, they struggle a lot because of their finances maybe " Key Informant 2*<br>*"Hygiene is a major issue in old persons generally I would say it's a major like I don't know some of them are staying alone, we have some jajjers who stay alone like there is no one then we have others children comes once in a while so hygiene is really an issue so when they get such illness it worsens*" **Key Informant 2**<br>*"… where they can be the one who are really where someone can take care of them and hygiene was the major issue would have our patients who would feel be okay if like taken care of by someone medical someone who knows what to do like hygiene and feeding. Its still in the pipeline but that was one of the major issues much as we give them, they are struggling and the caretakers are struggling*" **Key Informant 2**
	Support needed	Psychosocial support need by the informal caregivers	**Group therapy**<br>"*Well the emotional support that they need or psychological support I think would be group therapy just like I mentioned whenever they meet actually that is one thing we have notice when we meet them they become so happy remember they live in a very isolated place you have seen the distance between them so whenever they meet in one place and rejoice they dance their traditional dances they have preaching from evangelist, the health talk*

			most of them have been telling us that we even do it weekly so the group therapy I think heals them faster of course in addition to medications and other things" **Key Informant 3**<br>**Counselling**<br>"*Because they need counselling which I think we have tried to do in the room but I think its not enough since something that really goes on and on and these keepers are these and of course you see them stressed I don't know we just need to counsel them like keep on checking on them and counsel them*" **Key Informant 2**
		Other concerns	"*They have little questions because most of the things ROTOM provides like food, like jellies and soap because those are the things they would be asking but fortunately ROTOM sometimes give them those things and for psychosocial support ROTOM we have the evangelist of which they normally go and share their challenges about the stresses and what so they don't have a lot of questions*" **Key informant 1**<br><br>**Limited training on self care**<br>"*Well that one can be done when we of course now we faced challenges of finances normally what we do is we might not teach the care takers but we normally teach our field assistants but of late we have not been teaching about dementia care we have been teaching the things to do with non-communicable diseases of course dementia is also part but we have been focusing on hypertension, dementia, screening breast cancer all those thing we have not been very critical on that but we have challenge on finances we would love to call all the care takers but now we have a challenge of but we can give we have the finances we can be able to conduct those trainings*" **Key informant 3**

			**Negative Implication of cultural practices and tradition on care of dementia patients**<br>“*I think they worsen the prognosis because someone is already dealing with something else someone comes with cuts everywhere and what and now you are treating this and getting other infections so maybe for us as health workers they because we have received once in a while patients who come with cuts but not really dementia patients alone but patients with different other illnesses because they diagnose from the village their own then they bring here yet its something else so I think it limit on how better the patients will be* ” **Key Informant 2**
		Coping strategies	“*Yes, they tell them, the evangelist, to the field nurse and to the field implementor and it is through counselling and sharing with them that their problems can be solved or they can be relieved*” **Key informant 1**<br><br>**Evangelisation, Social workers**<br>“*That one is the part for the social worker, that is what social workers have been doing they have been engaging them, they have been carrying out fellowship where they go meet as elderly talk their prayers worshiping they sing so most of the time is done quarterly every three months this fellowship and then even these social workers we really engage them a lot during the field visit so that is the only way we have been helping them on that stress reaction the evangelist team has been doing this at the general fellowship which is conducted every quarter every three months so they have been meeting together and discussing from those fellowships we have had teachings from the health team, teachings from the evangelist teachings from the social workers*” **Key Informant 3**

		Strategies put in place to curb caregiver burden	**Community-based drug refills**<br>"*We normally get refills so what happens is the field nurse always make visit during her visit she goes with drugs but even before then we told those care takers that once their drugs are done they inform the field assistants who inform the field nurse and we have the channel of sending drugs to them we pack them level them and then send to them we have a field nurse to have some other people who take the drugs so that they don't come first of all we income security they don't have the money from kahaama to this is 20,000/ so 40,000/ is a lot of money every month so we make sure that we deliver the drugs.*" **Key informant 3**<br><br>**Advocating for social workers**<br>"*Yeah, we had much as we are health workers there are some skills we really lack though we taught psychology we are taught various things of course but I believe there specialized people that can do that work because like for us here we were thinking of having social work on the team not specifically dementia patient but we have many patients who are in the terminal illness. who are not doing well and sometimes for us we really lack the right words to use but we feel actually we have one who comes in but she is just helping she is still doing school she comes around but you find when she talks to these people and even these usual patients go and bathe the patient if you don't want to bathe she talks to them and the patient accepts to bath so social workers in health is really something yeah we need them on board*" **Key Informant 2**
Training	Training needs	Specific areas that need more training	Dealing with forgetfulness<br>"*It is that point of forgetfulness you know when you are staying with someone who always forget and most of the time interrupt things you may find they are*

			always complaining about how those people they are living with" **Key informant 1**<br>"*Maybe giving them a lot of trainings on hygiene, on the food they should give and to encourage them giving them their drugs they shouldn't stop, they shouldn't get tired of giving them their drugs*" **key informant 1**<br>"*One is drug compliancy, shouldn't get tired of giving them their drugs another thing is that on diet they should feed them like any other person and lastly is on the hygiene since sometimes they can't take care of themselves"* **key informant 1**<br><br>**Communication, Drug adherence, Self care**<br>"*Yeah of course the topic should be how to communicate with dementia patient, then teaching them on drug adherence, then would think of teaching them self-care yeah I would think of those* " **Key Informant 3**<br><br>**General care of dementia patients, Relating with dementia patients**<br>"*General care for the patient how they can like how they should relate with them and you know you can't tell a patient and tell their condition like how should they relate with them not to isolate them, not to take them as a burden I think they should be or some of them fear them, ok they don't really fear them but there is a way they take them like for you, you are there but you are in a home but you are there you are a special cases which is we should involve them in*" **Key Informant 2**<br><br>**Hygiene, Signs and Symptoms of dementia, Communication**

			“*Hygiene of course, feeding them, also we need to know when it’s not dementia because you know someone can have an illness they progress something else and for you are like its dementia and you leave the patient at home and we need to be trained on when to see that this is not the usual behavior of a patient and bring them to the hospital ”* Key Informant 2<br>*“Care, hygiene, feeding, how to relate or communicate with them, I don’t know what else*” Key Informant 2
		Support needed	Financial support<br>*“We could arrange maybe every year maybe twice a year we could make our small budget and probably I can also apply for the small grant but people like you who come here could lobby for us funds”* **Key Informant 3**
		Topics to be prioritised	“*Me I would prioritize drug adherence*” Key informant 1<br>“*Maybe also like they shouldn’t neglect these patients because they have dementia, they should have that relationship because that relationship that will also boost the patient*” Key informant 1
		Cultural beliefs and practices that may impact willingness to adopt new caregiving techniques	“*Since someone is believing bewitches you will find it hard that even being a care giver is giving the senior medication because if someone is believing in bewitches will take the senior to the witch doctors and churches that’s the problem....”* **Key informant 1**<br>“*So those new techniques since most of them will be thinking of bewitches or the spirit or the what because some think the spirits that are attacking them so I think it will be hard for someone who has that mind of bewitching to adopt a new system and the only way to help the care giver with such mind it is through sensitization to help someone with that mind otherwise if someone*

			has that mind and you don't sensitized that person you won't be doing anything" **key informant 1**<br>**"***Most of them feel attached to traditional that maybe there is something wrong with this person, so you find they are not helping or they even neglect I think of getting tired of taking care of but the thing is not going you find that they really neglecting them in helping them in day to day activities like any other chronic illness "* **Key Informant 2**<br><br>**Bewitching**<br>"*What I think is community still believe in things to do with superstitions this person has been bewitched I think somehow it burdens our care takers because care takers think that by believing normally they still to go to the witch doctor so you find them not believing in the drug that you are giving them in fact you find them not even giving the drugs so that eventually worsens the patients so I think the community also needs awareness so that in turn they our caretakers can really believe in the hospital remedies apart from the traditional remedies"* **Key Informant 3**<br><br>**Looking at dementia as a normal aging process rather than an illness**<br>*"For them they feel some know that it come with aging like how people be its not something that you should cause an alarm to go to hospital and take medication and be there after all other people were like this ate and then died so they don't take it serious so it would be that's what I said that most of them take it serous when it's like over time so and so is really not okay so I don't know what can be done I believe they can change, we can educate them"* **Key Informant 2**

	Training format	Delivery mode	**In-person workshop**<br>"*Like in ROTOM we call the care givers, we give them the time and place when to meet them but I think to non ROTOM places they should also go through churches and they health educate those people incase churches they put advert and what they also meet them give them date and time*" key informant 1<br>**Translated training manuals, Physical training, Case studies.**<br>*" Well what we normally do is what would be the best because when you talk of giving them trainers manual or something it would be difficult well you can do it but translate it in the language they understand when you give them a manual the care takers manual how they can take care of the but practically how to of course definitely call them case studies, physical trainings even case studies and then ask them actually how would you handle this so you involved them on how they would handle that I don't know how you call it in research language but I would think calling them physically and engaging them participating, workshops and then of course should be participatory, they should always participate, then of course designing a manual a simple manual translated in their language I think it would help them"* **Key Informant 3**<br><br>**In-person trainings based on experiences of the caregivers**<br>*"I think these people they have those need but they understand like if you speak to them you tell them the signs and symptoms and how they started and they can reason it with you and say by the way we started like this like she is saying I don't think they are really rigid because even when they come here and we tell them was it like this now they reason with you they know what you are asking you because that's how the patient started like this*

			behaving like this you find like they are willing to medical attention I don't think for me I don't but think it would really take a lot for them like you are telling them you are wrong as long as you tell them this is how the patient starts this is the age this is how I feel they are just you don't have the knowledge but once they get the knowledge they will adjust " **Key Informant 2**<br><br>Health talks<br>*"Health talk of course, maybe for us here we gather them in church fellowship we have those church fellowship they meet and praise God every month in different villages so it's always a perfect time to deliver information or you can also tell them that day you come with someone or someone you stay with of course some don't stay with anyone not even those who come alone they have intellect, they can click like this and this is being taught I think health talk in church older people are better gathered in churches for them you get them in church they come and pray, they sing they dance then talk to them"* **Key Informant 2**<br>**Workshops may not work in this region**<br>*" Workshop wont work in this region someone to come learn on dementia a care giver even those that have patients me I don't think it would really work but care giver those older person are well known by the church for me even when it's not here I feel like I don't know when they age they think they are going to die you find that the reverend knows them the fathers you find they are more attached to the church if the church call them they will easily respond or the church told them that on this day you are come with the person you stay with we have a program but to just come and leave the jaja*

			there and come like here to learn on taking care of the jaja it will be really hard " **Key Informant 2**<br>**Church meetings**<br>"*for me I think church is the perfect to gather those older people and their care givers because we tried it here but they respond to church their…basomesa of the village they know them very well*" Key Informant 2
		Other factors to consider	"*For us at ROTOM we don't have challenge with them because their transport is facilitated, their lunch is facilitated and for those who know how to read and write we give them the books and pens so that they can document something maybe the only challenge is that sometimes the season since they are the farmers they like cultivating you may find if it is a season of planting, you may get you back our caretakers they don't do that if you call them they come*" Key informant 1<br>"*Yeah, I think we should also consider those things especially the season, we see when it is maybe in the dry season when the activities concern with digging are limited but the only thing is that even if it is a season and you tell them the reason as to why you want to meet them, we may get good number still."* Key informant 1<br><br>Community outreach for mobilisation of informal caregivers<br>"*Its better we involve house to house, there are two ways you could actually go to the communities maybe you go to churches or schools invite them for a short time or you actually find them in their homes you deliver that teaching but otherwise if it involves them coming from their areas, it may be hard*" **Key Informant 3**

			**Workshops organised by ROTOM have a high chance of being a success**<br>*"There they can come knowing they expect something they know they will eat evening they go but coming alone but if through the organization they will respond they are used to you come you need to be talked to, they come but if it's just a random like Rukiga we have put a workshop maybe advertising it of course now people are the ones benefiting there I was seeing a gap for them to just come and advertise and some just come from their home to come and learn but if organization like here because they have their numbers and its easy"* **Key Informant 2**
