# Supplementary material for: Training needs of informal caregivers of persons with dementia in rural southwestern Uganda: A qualitative formative study
Source: Glob Ment Health (Camb). 2026 Jul 30;13:e171. doi: 10.1017/gmh.2026.10279 (PMC13458857; doi:10.1017/gmh.2026.10279)
Supplement: Peer review history

# Reviewer report: Review: Training needs of informal caregivers of persons with dementia in rural southwestern Uganda: A qualitative formative study — R0/PR2

**DOI:** 10.1017/gmh.2026.10279.pr2

Thank you for the opportunity to review this important study. Dementia and its related nuances are understudied in SSA. And so, any study that explores an aspect of it is welcomed. That said, some areas require further clarification and improvements.

Background

1. Lines 66-68: The citations supporting this sentence do not reflect global literature an Italian and UK literature. The authors are advised to check this.

2. “Globally, more than 5567 million people are currently living with dementia.” In which year? This should be indicated.

3. The first paragraph can be divided into two paragraphs. The first paragraph should focus on the epidemiology of dementia as already done. Then the second paragraph would then focus on informal caregivers, the stress they endure and coping mechanisms. As much as possible, literature should be drawn from SSA countries.

4. Before jumping into the WHO isupport framework, it is imperative to shed light on the dementia caregiving policy or framework in Uganda. How is it integrated in the healthcare system and in palliative care services? This should be clarified.

5. The paragraph on the WHO iSupport framework is poorly developed. It talks about why the tool was developed but fails to give details about what the tool constitutes and how it is expected to be used.

6. Lines 85-87: The authors indicated that the tool has been adapted across diverse settings. However, this is not explicitly explained. Describing this in detail will help other researchers in SSA to better appreciate this tool.

Methods

7. While the authors did indicate that caregiving is mostly rendered by family members, the methods section fails to explicitly indicate whether the informal caregivers in this study are purely family caregivers or other caregivers in informal settings. This should be clarified.

8. What was the stopping criterion, and how was it established that informational saturation had been reached?

9. More details about the data collection will be great. Who were the research assistants? How many were they?

10. How was rigor, trustworthiness, and reflexivity ensured in this study?

11. Include a the coding book or coding tree to show how the codes evolved to themes.

Results and discussion

12. The results are insightful; however, the presentation of it is a bit problematic. In qualitative research, the descriptors of the findings should be thick and detailed.

13. Some of the extracted quotes don’t match the theme. E.g., “It is my burden to die with.” Caregiver 5, Male, 75 years. I fail to see how this is a coping strategy.

14. The findings are too condensed. There should be sub-themes to allow the authors to be effectively describe the findings.

15. Include a section under the discussion to highlight the implications for policy, practice and future studies.

# Reviewer report: Review: Training needs of informal caregivers of persons with dementia in rural southwestern Uganda: A qualitative formative study — R0/PR3

**DOI:** 10.1017/gmh.2026.10279.pr3

This is a well-written manuscript bringing evidence on a very important topic. A few minor comments to the authors:

In the introduction, I suggest not describing “dementia as a silent neurodegenerative condition”. I suggest using standard definitions for dementia

In the introduction-the reference given for this sentence “Globally, more than 55 million people are currently living with dementia, a number projected to nearly double every 20 years……”, I suggest including this as part of the references https://www.alzint.org/about/dementia-facts-figures/dementia-statistics/

Please cite the WHO iSupport reference in the introduction: https://www.who.int/publications/i/item/9789241515863

Only 3 key informants were included in the interviews. Did you also reach saturation with just these three key informants? Could you provide details on how the findings were combined even though the interview guides were different?

Can you also explain if there were differences between what the caregivers and the key informants said?

In the discussion section- “Our results indicate that most caregivers in rural Uganda attribute dementia…..” please specify that it is caregivers who were interviewed so that it is not a generalisable statement.

# Reviewer report: Review: Training needs of informal caregivers of persons with dementia in rural southwestern Uganda: A qualitative formative study — R0/PR4

**DOI:** 10.1017/gmh.2026.10279.pr4

Thank you for the opportunity to review this manuscript. The topic is timely and relevant, and the authors address an important issue in dementia care with a low resourced and rural setting. The manuscript is well written and mostly flows well. Below I provide comments intended to strengthen the clarity, rigour and contribution of the work.

Background

1. The background sets up the need for the study well and appropriately describes background relevant to the study setting. The references appear to be a little out of order or may be incorrect. Please check the first few references to make sure they support the statements made in the introduction. In particular, reference 16 is cited when referencing the WHO iSupport tool - please check if this is the most appropriate reference.

Study population, sample size and sampling

2. Line 120 - It would be helpful to clarify here that the informal caregivers are clients of ROTOM. Currently, this information doesn’t become clear until later on in the manuscript. It would also be helpful to identify in this section that the key informant are health professional staff members at ROTOM.

3. The manuscript describes how participants were selected however information about how they were invited to participate is missing. Additionally, further information about how and why participants were selected from the database is needed.

Data management and analysis

4. What methodology guided the thematic analysis? For example, Braun and Clarke

5. How did the authors go about synthesising and interpreting the different stakeholder views?

Findings

6. Line 256-259. Further information is needed here. I understand that this is a key informant view however it isn’t clear how this integrates with the theme.

7. The authors' highlight that the participant group represents a wide variety of views. Were there any particular differences or similarities in views or experiences when considering the situations of each participant and the strengths they had access to or challenges they faced?

Discussion

7. Given that this is formative research, could the authors consider including a section about future research or next steps?

# Editor report: Recommendation: Training needs of informal caregivers of persons with dementia in rural southwestern Uganda: A qualitative formative study — R0/PR5

**DOI:** 10.1017/gmh.2026.10279.pr5

**Affiliations:** London School of Hygiene & Tropical Medicine, United Kingdom of Great Britain and Northern Ireland

This is an important study however there are a number of issues needing to be addressed before it can be considered for publication as highlighted by the three reviewers.

# Editor report: Decision: Training needs of informal caregivers of persons with dementia in rural southwestern Uganda: A qualitative formative study — R0/PR6

**DOI:** 10.1017/gmh.2026.10279.pr6

**Affiliations:** London School of Hygiene & Tropical Medicine, United Kingdom of Great Britain and Northern Ireland

No accompanying comment.

# Reviewer report: Review: Training needs of informal caregivers of persons with dementia in rural southwestern Uganda: A qualitative formative study — R1/PR8

**DOI:** 10.1017/gmh.2026.10279.pr8

The authors have answered the questions satisfactorily. I have no further comments.

# Reviewer report: Review: Training needs of informal caregivers of persons with dementia in rural southwestern Uganda: A qualitative formative study — R1/PR9

**DOI:** 10.1017/gmh.2026.10279.pr9

Dear authors,

Congratulations on this revised manuscript. The changes made significantly improve the contribution of this study to the evidence base. I am satisfied that my comments, and those of other reviewers, have been addressed. I look forward to seeing this in published from.

# Editor report: Recommendation: Training needs of informal caregivers of persons with dementia in rural southwestern Uganda: A qualitative formative study — R1/PR10

**DOI:** 10.1017/gmh.2026.10279.pr10

**Affiliations:** London School of Hygiene & Tropical Medicine, United Kingdom of Great Britain and Northern Ireland

The manuscript has been revised sufficiently to meet journal requirements for publication

# Editor report: Decision: Training needs of informal caregivers of persons with dementia in rural southwestern Uganda: A qualitative formative study — R1/PR11

**DOI:** 10.1017/gmh.2026.10279.pr11

**Affiliations:** London School of Hygiene & Tropical Medicine, United Kingdom of Great Britain and Northern Ireland

No accompanying comment.
